# A panoramic view of cotton resistance to *Verticillium dahliae*: From genetic architectures to precision genomic selection

**DOI:** 10.1002/imt2.70029

**Published:** 2025-04-11

**Authors:** Xiaojun Zhang, Shiming Liu, Peng Wu, Wanying Xu, Dingyi Yang, Yuqing Ming, Shenghua Xiao, Weiran Wang, Jun Ma, Xinhui Nie, Zhan Gao, Junyuan Lv, Fei Wu, Zhaoguang Yang, Baoxin Zheng, Ping Du, Jiangmei Wang, Hao Ding, Jie Kong, Alifu Aierxi, Yu Yu, Wei Gao, Zhongxu Lin, Chunyuan You, Keith Lindsey, Nataša Štajner, Maojun Wang, Jiahe Wu, Shuangxia Jin, Xianlong Zhang, Longfu Zhu

**Affiliations:** ^1^ National Key Laboratory of Crop Genetic Improvement Huazhong Agricultural University Wuhan China; ^2^ Hubei Hongshan Laboratory Wuhan China; ^3^ Institute of Economic Crops Xinjiang Academy of Agricultural Sciences Urumqi China; ^4^ College of Agriculture Shihezi University Shihezi China; ^5^ Cotton Research Institute Xinjiang Academy of Agriculture and Reclamation Science Shihezi China; ^6^ State Key Laboratory of Cotton Biology Henan University Kaifeng China; ^7^ Department of Biosciences Durham University Durham UK; ^8^ Biotechnical Faculty University of Ljubljana Ljubljana Slovenia; ^9^ State Key Laboratory of Plant Genomics, Institute of Microbiology Chinese Academy of Sciences Beijing China

**Keywords:** armadillo‐repeat protein, cotton, genomic selection, genome‐wide association studies, ROS homeostasis, transcriptome‐wide association studies, Verticillium wilt

## Abstract

Investigating the genetic regulatory mechanisms underlying complex traits forms the foundation for crop improvement. Verticillium wilt (VW), caused by *Verticillium dahlia*e (*V. dahliae*), is one of the most devastating diseases affecting crop production worldwide. However, the genetic basis underlying crop resistance to *V. dahliae* remains largely obscure, hindering progress in the genomic selection for VW resistance breeding. Here, we unraveled the genetic architectures and regulatory landscape of VW resistance in cotton by combining genome‐wide association studies (GWAS) and transcriptome‐wide association studies (TWAS) using 1152 transcriptomes derived from 290 cotton accessions. We identified 10 reliable quantitative trait loci (QTLs) associated with VW resistance across multiple environments. These QTLs showed a pyramiding resistance effect and exhibited promising efficacy in the genomic prediction of cotton's VW resistance supported by an F_2:3_ population. Moreover, trace analysis of these elite alleles revealed a notably increased utilization of Lsnp1, Lsnp4, Lsnp5, Lsnp8, and Lsnp9, which potentially contribute to the improvement of VW resistance in Chinese cotton breeding since the 1990s. We also identified remarkable gene modules and expression QTL (eQTL) hotspots related to the regulation of reactive oxygen species (ROS) homeostasis and immune response. Furthermore, 15 candidate causal genes were prioritized by TWAS. Knocking down eight genes with a negative effect significantly enhanced cotton resistance to *V. dahliae*. Among them, *GhARM*, encoding an armadillo (ARM)‐repeat protein, was verified to modulate cotton resistance to *V. dahliae* by regulating ROS homeostasis. Overall, this study updates the understanding of the genetic basis and regulatory mechanisms of cotton's VW resistance, providing valuable strategies for VW management through genomic selection in cotton breeding.

## Highlights

- Ten reliable quantitative trait loci governing cotton resistance to *V. dahliae* in various environments were identified through a genome‐wide association studies (GWAS) using an upland cotton population comprising 290 accessions. The pyramid of these 10 elite alleles reduced the disease index of cotton to *V. dahliae* from 70 to 20.
- Trace analysis of these elite alleles revealed that the increased utilization of Lsnp1^R^, Lsnp4^R^, Lsnp5^R^, Lsnp8^R^, and Lsnp9^R^ since the 1990s aligned with Chinese cotton breeding programs, offering new insights into interpreting the advancements in cotton breeding for Verticillium wilt resistance in China.
- Analysis of 1152 transcriptomes under normal and *V. dahliae*‐stress conditions elucidated the regulatory network governing cotton's response to *V. dahliae*, highlighting the regulation of reactive oxygen species (ROS) homeostasis and immune response.
- By combining GWAS and transcriptome‐wide association studies, 15 candidate causal genes were pinpointed, with *GhARM* identified as a negative regulator of cotton resistance to *V. dahliae* through the modulation of ROS homeostasis.

## Graphical abstract

This study identified 10 reliable quantitative trait loci (QTLs) governing cotton resistance to *V. dahliae* in various environments through a genome‐wide association analysis (GWAS) using an upland cotton population. The pyramid of these 10 elite alleles reduced the disease index of cotton to *V. dahliae* from 70 to 20. Trace analysis of these elite alleles revealed that the increased utilization of Lsnp1^R^, Lsnp4^R^, Lsnp5^R^, Lsnp8^R^, and Lsnp9^R^ since the 1990s aligned with Chinese cotton breeding programs, offering new insights into interpreting the advancements in cotton breeding for Verticillium wilt resistance in China. Analysis of 1152 transcriptomes under normal and *V. dahliae*‐stress conditions elucidated the regulatory network governing cotton's response to *V. dahliae*, highlighting the regulation of reactive oxygen species (ROS) homeostasis and immune response. By combining genome‐wide association analysis and transcriptome‐wide association studies, 15 candidate causal genes were pinpointed, with *GhARM* identified as a negative regulator of cotton resistance to *V. dahliae* through the modulation of ROS homeostasis.

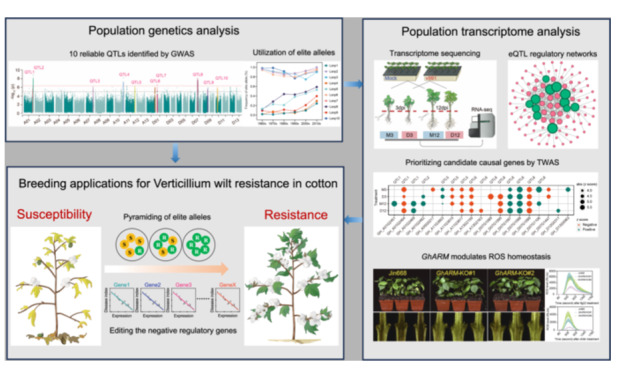

## INTRODUCTION

Cotton is an important crop, providing 35% of the natural fibers for the textile industry in the world [1]. Verticillium wilt (VW), caused by *Verticillium dahliae* (*V. dahliae*), is a highly destructive soil‐borne disease that can affect over 200 plant species, including cotton, tomato, potato, olive, and hop, resulting in yield reductions of 10%–60% and significant economic loss [2]. VW causes 10%–35% yield losses in cotton production worldwide, with over 40% of cotton acreage affected in China, resulting in an economic loss of 250−310 million dollars annually [3]. Moreover, *V. dahliae* persists and accumulates as microsclerotia in the soil, increasing the incidence of VW in cotton through stubble return and continuous monocropping [4]. The use of VW‐resistant varieties is the most efficient disease management.

The complexity of genetics determines the challenges of improving quantitative traits, especially in plant disease resistance; the resistance of varieties harboring the single resistance gene is easily broken through by pathotypes. Genetic analysis uncovers that the resistance to *V. dahliae* race1 in tomato is governed by a dominant gene, *Ve1*, while cotton, eggplant, and potato exhibit the typical quantitative trait inheritance for resistance to *V. dahliae* [5, 6, 7, 8]. Through genome‐wide association studies (GWAS), some loci associated with VW resistance on chromosomes A10 and D11 have been identified [9, 10, 11]. Additionally, virus‐induced gene silencing (VIGS) has uncovered a few of genes regulating resistance to *V. dahliae* in cotton, such as *CG02* encoding NBS‐LRR [10], *GhRVD1* encoding Toll/Interleukin 1 receptor (TIR) NLR (TNL) [12], and *GhNCS* encoding (S)‐norcoclaurine synthase [9]. However, for the complex quantitative traits of cotton resistance to *V. dahliae*, whole genome identification and evolutionary analysis of VW resistant loci are crucial for breeding disease‐resistant cotton.

In human, the Genotype‐Tissue Expression (GTEx) project provides a comprehensive catalog of genetic variants and their impact on gene expression levels in different tissues [13]. Researchers can use transcriptome‐wide association studies (TWAS) to integrate expression quantitative trait loci (eQTL) data from GTEx with GWAS data to identify genes associated with complex diseases, thus gaining insights into the regulatory mechanisms underlying disease development [14]. Moreover, the application of eQTLs and TWAS analyses has recently expanded to encompass a wide range of crops. For instance, in maize, these analyses have been used to understand the genetic regulation of complex agronomic traits such as drought tolerance, heat stress, and adaptation to temperate environments [15, 16, 17, 18, 19]. Studies on *Brassica napus* have focused on seed oil content [20, 21, 22, 23], while rice investigations have explored panicle architecture and salt tolerance [24, 25]. In cotton, the main focus has been on fiber quality [26, 27, 28], with limited studies on high‐temperature stress [29] and cotton seed yield [30]. Nonetheless, there is currently a lack of eQTL studies on the genetic regulation of cotton resistance to *V. dahliae*. By prioritizing candidate genes associated with the trait of interest within GWAS‐identified genomic regions, TWAS offers a comprehensive understanding of the genetic regulation.

Over the past 50 years, continuous studies have been conducted worldwide on the genetic basis and breeding for cotton resistance to *V. dahliae*. Although the resistance of varieties has been improved, the development of highly resistant varieties remains a challenging endeavor in breeding programs. To enhance understanding of the genetic basis and regulatory mechanisms of VW resistance in cotton and advance genomic selection for VW‐resistant cotton breeding. We combined GWAS, TWAS, and gene co‐pression modules analysis to identify the QTLs, candidate genes, and regulatory networks associated with cotton resistance to *V. dahliae* based on a genomic variation map and 1152 transcriptomes derived from 290 cotton accessions under both normal and *V. dahliae*‐stress conditions. Candidate genes prioritized by multi‐omics analyses were experimentally validated through gene knockdown and knockout assays to confirm their roles in VW resistance. This study shed light on the genetic basis and regulatory mechanisms underlying cotton's VW resistance, and provides valuable insights for genomic selection in cotton breeding and genetic improvement of complex traits.

## RESULTS

### GWAS reveals the intricate genetic architecture underlying resistance to *V. dahliae* in upland cotton

**Figure 1:**
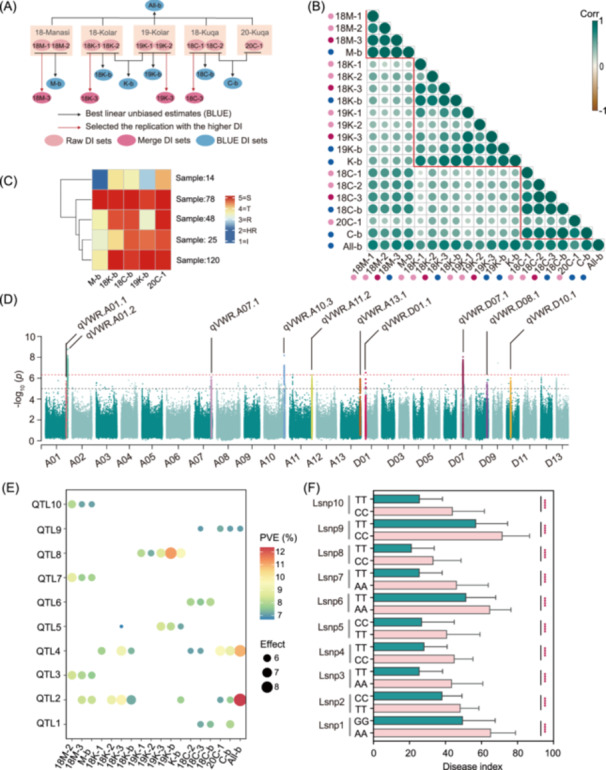
Phenotypic analysis and genome‐wide association studies (GWAS) of cotton's VW resistance at different environments. (A) Introduction to disease index (DI) and processing workflow. The pink, dark red, and blue circles represent the raw, merged, and BLUE DI sets. The labels inside the circles are the names of the DIs, the naming principle is: year‐location‐data type, 18, 19, and 20 represent the years 2018, 2019, and 2020, respectively. The capital letters M, K, and C represent the locations Manasi, Kolar, and Kuqa, respectively. The terminal numbers 1, 2, 3, and letter b represent replication1, replication2, merge DI, and BLUE DI, respectively. (B) Correlation (Corr) analysis of 20 DI sets. The pink, dark red, and blue circles labels represent the raw, merged, and BLUE DI sets. The circle size indicates the PCC value. (C) Cluster heatmap of DI in five independent environments. The numerical scale from 1 to 5 represents immune (I), highly resistant (HR), resistant (R), tolerant (T), and susceptible (S), respectively. The sample number represents the count of cotton accessions belonging to the cluster. (D) Manhattan plot of GWAS for 20 DI sets. Quantitative trait loci (QTLs) identified at least three times are labeled. The *x*‐axis shows the 26 chromosomes (A01–A13 and D01–D13) in *G. hirsutum*. The *y*‐axis represents the –log_10_ (*p*) of SNPs from the GWAS. Significance thresholds of *p* = 4.65E‐05 (1/*n*, *n* is the total number of SNPs) and *p* = 1E‐05 (experience‐based). (E) The percentage of PVE and effects of 10 QTLs. A circle indicates a significant correlation between this QTL and the phenotype in this group. The circle size indicates the effect. The circle color indicates the percentage of PVE. (F) Distribution of DI with two different genotypes of Lsnp of 10 QTLs. Two‐sided Student's *t*‐test, ****p < 0.0001. Column height represents the mean, and error bars represent ± SD. BLUE, best linear unbiased estimates; PCC, Pearson correlation coefficient; PVE, phenotypic variance explained; VW, Verticillium wilt.

A natural population of 290 upland cotton accessions previously subjected to re‐sequencing [31] (Table S1), was evaluated over 3 years in VW nurseries in Manasi, Korla, and Kuqa in China. Nine raw disease index (DI) sets were obtained in five independent environments (18‐Manasi, 18‐Korla, 18‐Kuqa, 19‐Kolar, and 20‐Kuqa) (Figure 1A and Figure S1). The frequency distribution of these raw DI sets exhibited a normal distribution (Figure S2). By performing the best linear unbiased estimates (BLUE) calculations on the raw DI sets, seven BLUE DI sets were obtained, showing a strong correlation with the raw DI sets (Figure 1A and Figure S3). Additionally, by merging DI datasets based on the maximum values from two replicates, four merged DI sets were obtained. In total, 20 sets of DI data were generated (Figure 1A and Table S2). Pearson correlation coefficients (*r*) from the same site (0.33–0.67) were higher than those from the same year (0.13–0.63) (Figure 1B), suggesting that the environment at the different sites is an important determinant of accession resistance. The cotton accessions could be classified into five clusters using the K‐means clustering method based on their DIs in the five independent environments (Figure 1C and Figure S4). Among them, 78 accessions (27.4%) consistently exhibited susceptibility in all environments, while 14 accessions (4.9%) showed consistent resistance across the five environments (Figure 1C). Disease resistance of 67.7% of accessions was unstable and susceptible in at least three environments (Figure 1C). This finding emphasizes that the environment has a significant impact on disease incidence and that the predominant germplasm for cotton resistance is hardly observed in China.

We conducted a GWAS based on the mixed linear model in GEMMA with 20 sets of DI using 2,152,153 SNPs (Figure S5). A total of 22 significantly associated loci were identified across different environments over multiple years, covering 15 chromosomes, with 13 loci in the At subgenome and 9 ones in the Dt subgenome (Figure 1D and Table S3). Among the 22 loci, 10 were consistently detected in at least three DI sets, with 6 being environmentally stable loci and 4 being environmentally specific loci, defining them as VW‐resistant QTLs (Figure 1D, Tables 1 and S3). For each QTL, the SNP with the most significant *p‐*value was designated as Lead snp1 to Lead snp10 (Lsnp1–Lsnp10). The phenotypic variation explained by Lsnp1–Lsnp10 ranged from 7.21% to 12.36%, showing a highly significant correlation with the DI (Figure 1E,F).

**Table 1:** Summary of 10 QTLs associated with VW resistance in cotton.

QTL‐ID	Name	Lead SNP	*p‐*Value	PVE (%)	Frequency	R allele	S allele
QTL1	*qVWR.A01.1*	A01_111929609	9.16E‐07	7.99	3	G	A
QTL2	*qVWR.A01.2*	A01_117983536	1.56E‐09	12.36	9	C	T
QTL3	*qVWR.A07.1*	A07_90971603	5.18E‐07	8.40	3	T	A
QTL4	*qVWR.A10.3*	A10_108893925	7.14E‐09	10.96	8	T	C
QTL5	*qVWR.A11.2*	A11_119799615	5.15E‐07	8.48	4	C	T
QTL6	*qVWR.A13.1*	A13_105434827	9.14E‐07	8.02	3	T	A
QTL7	*qVWR.D01.1*	D01_1696495	2.49E‐07	8.88	3	T	A
QTL8	*qVWR.D07.1*	D07_13371656	8.87E‐09	11.19	5	T	C
QTL9	*qVWR.D08.1*	D08_61445110	3.63E‐06	7.21	4	T	C
QTL10	*qVWR.D10.1*	D10_22542365	9.44E‐07	8.03	3	T	C

Abbreviations: QTLs, quantitative trait loci; VW, Verticillium wilt.

### **Genetic architecture of 10 Lsnp** ^R^ **s in Chinese cultivars**

**Figure 2:**
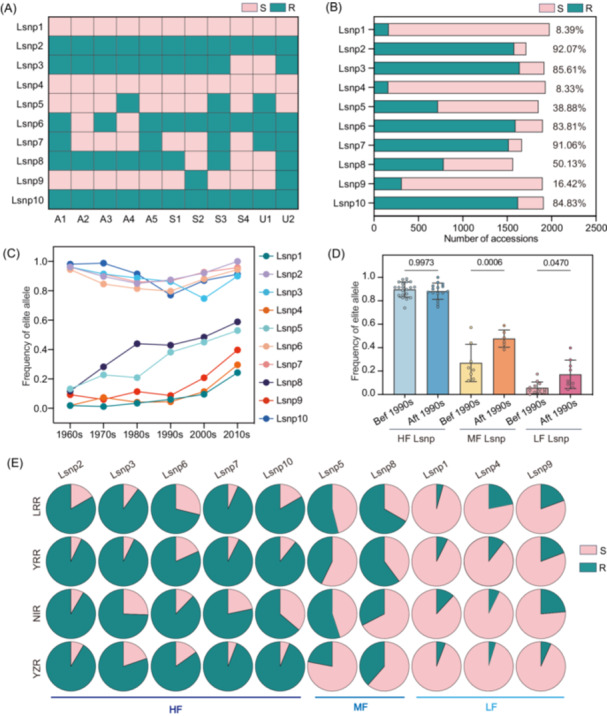
The origin, distribution, and evolution of 10 Lsnp^R^s. (A) Origin of 10 Lsnp^R^s analyzed based on foreign ancestors. A1–A5 represents American ancestors, S1–S3 represents Soviet ancestors, and U1–U2 represents Ugandan ancestors. Green represents Lsnp^R^ and pink represents Lsnp^S^. (B) Frequency of 10 Lsnp^R^s in the CDCs. Green represents Lsnp^R^ and pink represents Lsnp^S^. The numbers to the right of the column represent the utilization of Lsnp^R^s in CDCs. The *x*‐axis shows the number of accessions. (C) The utilization frequency of 10 Lsnp^R^s in cultivars developed at different time periods in China. The *x*‐axis shows the different time periods. The *y*‐axis shows the frequency of 10 Lsnp^R^s. (D) The difference in utilization frequency of 10 Lsnp^R^s before and after the 1990s. The *y*‐axis shows the frequency of 10 Lsnp^R^s. Two‐sided Student's *t*‐test. (E) The distribution of 10 Lsnp^R^s in the four major cotton‐growing regions in China. Green represents Lsnp^R^ and pink represents Lsnp^S^. Lsnp, lead SNP; Lsnp^R^, resistance (R) allele of the Lsnp; Lsnp^S^, susceptibility (S) allele of the Lsnp; CDCs, Chinese descendible cultivars; HF, high frequency; MF, moderate frequency; LF, low frequency.

The upland cotton germplasms in China were mainly introduced from the United States (USA) (such as King, Delfos 531C, DPL15, STV2B, and Trice), previous Soviet Union (SU) (such as 611B, KK1543, 108Φ, and C1470), and Uganda (UG) (such as UgandaMian3 and UgandaMian4) in 20th century and serve as the ancestral elites for cotton breeding [32]. To better understand the history and effectiveness of disease‐resistant breeding in China, elite alleles were identified among these 11 ancestors (Figure 2A). The results show that the distribution of 10 Lsnp^R^s (Lsnp^R^ represents resistance (R) allele of the Lsnp) can be classified into three types: the United States/Soviet Union/Uganda (ASU), the Soviet Union/Uganda (SU), and Others (Figure 2A). Among them, Lsnp2^R^, Lsnp3^R^, Lsnp5^R^, Lsnp6^R^, Lsnp7^R^, Lsnp8^R^, and Lsnp10^R^ could be found in the ancestors from the ASU, Lsnp9^R^ could only be found in SU (Figure 2A). While Lsnp1^R^ and Lsnp4^R^ could not be detected in these varieties, possibly originating from other ancestors or the breeding processes (Figure 2A). To explore the genetic architecture of these 10 Lsnp^R^ in cotton breeding, a total of 2033 nonredundant Chinese descendible cultivars (CDCs) were collected [26, 33, 34, 35, 36], including 1006 CDCs with release date and 1885 CDCs with geographical distributions (Figure S6A,B and Table S4). The results indicate that the widespread Lsnp2^R^, Lsnp3^R^, Lsnp6^R^, Lsnp7^R^ and Lsnp10^R^ in this elite have a consistently high frequency (HF) in CDCs (Figure 2A,B). However, in CDCs, the frequency of the common Lsnp8^R^, which is popular among ancestors is comparable to the moderate frequency (MF) of the Lsnp5^R^, which is rare among ancestors (Figure 2A,B). The elite alleles Lsnp1^R^, Lsnp4^R^ and Lsnp9^R^ exhibit low frequencies (LF) of 8.39%, 8.33%, and 16.42% in CDCs, respectively (Figure 2A,B). These observations indicate that Lsnp1^R^, Lsnp4^R^, Lsnp5^R^, Lsnp8^R^, and Lsnp9^R^ have not been widely used, revealing that only limited sources of resistance genes have been introduced in the past.

We also analyzed the utilization history of 10 Lsnp^R^ in CDCs. The results showed that HF Lsnp^R^ (Lsnp2^R^, Lsnp3^R^, Lsnp6^R^, Lsnp7^R^, and Lsnp10^R^) were popularly utilized with high frequency from 73.91% to 100% in all released varieties in history (Figure 2C). The utilization frequency of Lsnp1^R^, Lsnp4^R^, Lsnp5^R^, Lsnp8^R^, and Lsnp9^R^ increased more than 3 times from 1960s to 2010s. The usage of Lsnp5^R^ and Lsnp8^R^ increased from 11.76% in 1960s to 58.82% in 2010s, while the usage of Lsnp1^R^, Lsnp4^R^, and Lsnp9^R^ remains less than 40% even in 2010s (Figure 2C). Notably, the utilization of MF and LF Lsnp^R^ gradually increased from 1990s (Figure 2C,D). Additionally, we analyzed the geographic distribution pattern of Lsnp^R^ by classifying the 1885 CDCs into four major cotton production regions in China: Liao River region (LRR) with 71 CDCs, Yellow River region (YRR) with 946 CDCs, Yangtze River region (YZR) with 527 CDCs, and Northwest region (NIR) with 341 CDCs (Figure S6B). The results showed that the frequency variation range of MF Lsnp5^R^ and Lsnp8^R^ was highest in China, with the lowest frequency in YZR (Figure 2E). Overall, the utilization frequency of Lsnp^R^ was highest in LRR varieties (59.7%) and lowest in YZR varieties (52.21%) (Figure 2E). All these results indicate that the LRR and YRR varieties have a higher tolerance to *V. dahliae*. Moreover, the practice of water and drought rotation prevalent in YZR limits the continuous accumulation of pathogens. Consequently, cotton varieties in LRR and YRR, which are subject to greater selection pressure from the disease incidence, showed better disease resistance (Figure S7).

### Pyramiding effect of 10 Lsnp^R^s

**Figure 3:**
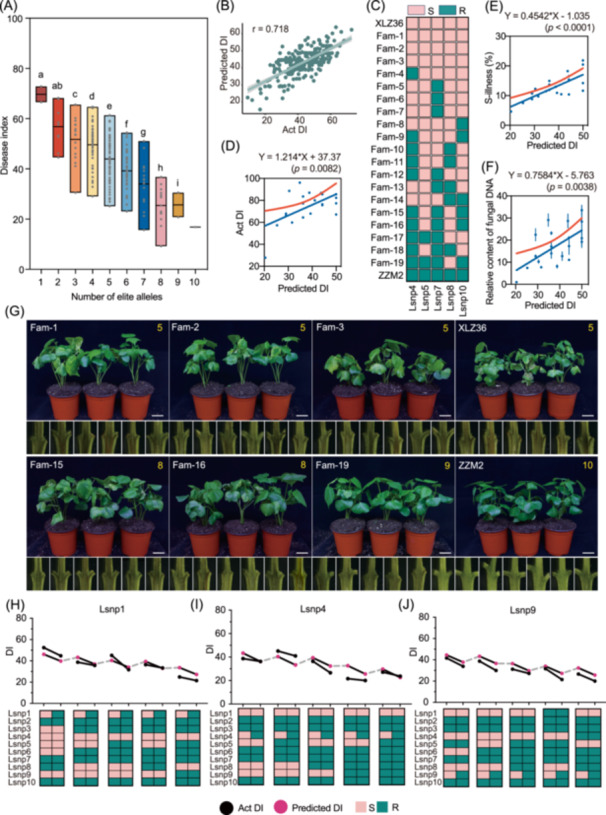
Pyramiding effect of 10 Lsnp^R^s. (A) Distribution of DI in cotton accessions carrying different numbers of Lsnp^R^. The *x*‐axis represents cotton accessions carrying 1–10 Lsnp^R^. The *y*‐axis represents All‐b disease index (DI) across all environments. The letters indicate the statistical test after the *t*‐test (*p* < 0.001). (B) The correlation between the DI predicted by the MDIC and the actual DI. The *x*‐axis represents predicted DI; the *y*‐axis represents the actual DI of the corresponding genotype materials. (C) The genotypes of 19 F_2_ individuals at 5 Lsnps. ZZM2 and XLZ36 are resistant and susceptible parents, respectively. (D) Correlation between actual DI and predicted DI in 19 F_2:3_ lines. (E) Correlation between extracted proportion of diseased area in stem sections and predicted DI in 19 F_2:3_ lines. (F) Correlation between actual vascular pathogen content and predicted DI in 19 F_2:3_ lines. D–F are all simple linear regressions, and the *p*‐value represents the hypothesis that the slope is non‐zero. (G) Six extreme F_2:3_ lines were selected from the predicted DI, with images showing the disease phenotype and cross‐sections of stems at leaf nodes. Photographed at 13 days post‐inoculation. The yellow number in the top right corner indicates the number of Lsnp^R^ carried by the line. Fam1–Fam3 are lines with the highest predicted DI. Fam15, 16, and 19 are lines with the lowest predicted DI. Scale bar, 3 cm. (H–J) The effect of enhancing VW resistance in existing cotton materials after transformation from Lsnp^S^ to Lsnp^R^. (H–J) Represents the DI distribution between the Lsnp^S^ series cotton varieties and Lsnp^R^ series cotton varieties at Lsnp1, 4, and 9 with low frequency. The genotypic map (below *x*‐axis) shows haplotypes across accessions, with the upper line graph indicating mean DI per haplotype. The black dots represent the actual DI, and the magenta dots represent the predicted DI by the MDIC. BLUE, best linear unbiased estimates; MDIC, molecular disease index calculator.

To validate the pyramiding effect of the 10 Lsnp^R^s, the accessions were first divided into 10 classes based on the Lsnp^R^s carried (Figure 3A), and then the pyramiding status was assessed. Overall, 71% and 71.8% of cotton varieties carried 4–6 Lsnp^R^, respectively (Figure S8A,B, Tables S4 and S5), and the number of Lsnp^R^s carried correlated positively with the disease resistance of the cotton (Figure 3A). It is noteworthy that Zhongzhimian2 (ZZM2), designated as the national regional trial VW‐resistant control variety [37], carried all 10 Lsnp^R^s and showed stable resistance in different environments (Figure S8C). Furthermore, five accessions (Jizi 1, Jizi 96, Xinluzhong 39, Xinluzhong 40, and Renhe39) within the 2033 CDCs carried all 10 Lsnp^R^s (Table S4). Consistent results were obtained using VW resistance data from published natural population, confirming the validity of the pyramiding effect in cotton accessions (Figure S8D,E). Furthermore, the effects of each Lsnp^R^ were estimated using a ridge regression model, which enabled the development of a molecular disease index calculator (MDIC). The predicted DI for different genotypic combinations correlated well with the observed DI (correlation coefficient of 0.718) (Figure 3B), indicating that these 10 Lsnps are predictive. Notably, three Lsnp^R^s, including Lsnp1^R^, Lsnp4^R^, and Lsnp9^R^ with low utilization frequencies, demonstrate pronounced effects, suggesting significant potential for targeted improvement of cotton resistance to *V. dahliae* through the pyramiding of these Lsnp^R^s (Figure S9).

To further validate the pyramiding effect, an F_2:3_ population of 272 families was constructed using ZZM2 and Xinluzao 36 (XLZ36, a susceptible accession) as parents (Figure S10). ZZM2 carries 10 Lsnp^R^, while XLZ36 has only Lsnp1^R^, Lsnp2^R^, Lsnp3^R^, Lsnp6^R^, and Lsnp9^R^. The genotypes of these 272 lines at the five Lsnp (Lsnp4, Lsnp5, Lsnp7, Lsnp8, and Lsnp10) were determined through sequencing (Figure S11). Among them, 42, 15, 64, 59, and 62 F_2_ individuals were homozygous at Lsnp4^R^, Lsnp5^R^, Lsnp7^R^, Lsnp8^R^, and Lsnp10^R^, respectively (Table S6). 19 F_2:3_ lines, demonstrating homozygous genotypes at all five Lsnp and exhibiting 13 different genotype combinations, were selected for subsequent inoculation assay (Figure 3C and Table S7). The results indicated that F_2:3_ lines with lower predicted DI had lower actual DI, smaller proportions of disease lesion area, and lower fungal biomass, indicating that lines pyramiding more Lsnp^R^s were more resistant to *V. dahliae* (Figures 3D−G and S12, Table S7). Additionally, we examined the actual impact of individual site in resistance improvements using published data [12]. We selected a pair of cotton varieties that differed solely at the Lsnp1, representing resistant and susceptible genotypes, respectively (Figure 3H). A comparison of DIs between these two series of cotton varieties revealed that the R‐genotype series at Lsnp1 exhibited lower DI than the S‐genotype series (Figure 3H). Similar validation results were obtained for the other eight Lsnps (except for Lsnp2, which was not detected through our calling SNP process) (Figures 3H−J and S13, Table S8). In conclusion, we validated the pyramiding effect of the 10 Lsnp^R^s using multiple natural populations and an artificial population, and demonstrated that the resistance could be significantly improved by pyramiding more Lsnp^R^s.

### Genes responsive to *V. dahliae* are mainly involved in reactive oxygen species (ROS) homeostasis and immune response

**Figure 4:**
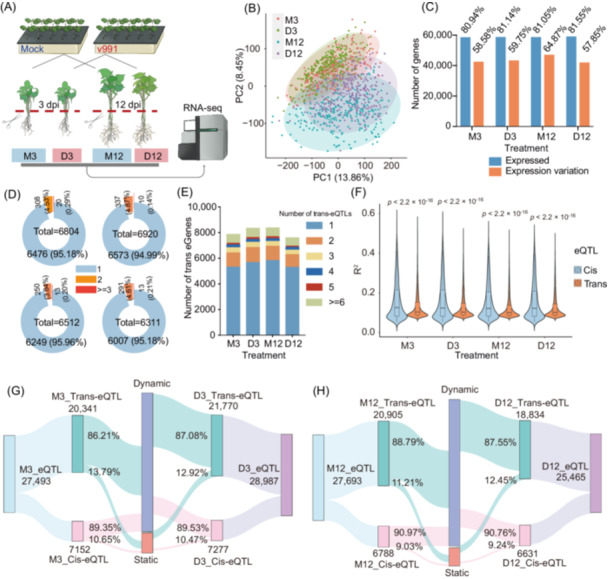
Overview of gene expression and expression quantitative trait loci (eQTL) atlas for cotton during early and late infection stage of *V. dahliae*. (A) Illustration of the treatment of *V. dahliae* and mock. Cotton root and about 1 cm cotyledon were collected at two time points for RNA‐seq: 3 days and 12 days after inoculation and mock treatment. Treatments are labeled as M3, D3, M12, and D12. (B) Principal component analysis dimensionality reduction plot of the 1152 transcriptomes. Each point represents a sample. (C) Statistic of the number of genes that expressed and the number of genes that exhibit expression variation. (D) The number of eGenes with different number of independent cis‐eQTL. (E) The number of eGenes with different number of trans‐eQTL. (F) Comparison of the explained variance (*R* ^2^) between cis‐eQTLs and trans‐eQTLs (two‐sided Wilcoxon rank sum test; center line, median; box limits, first and third quartiles). (G) Static and dynamic distribution of cis and trans‐eQTL in M3 and D3. (H) Static and dynamic distribution of cis‐ and trans‐eQTL in M12 and D12. M3, 3 days after mock treatment; D3, 3 days post‐inoculation; M12, 12 days after mock treatment; D12, 12 days post‐inoculation.

To assess the transcriptional response to *V. dahliae* stress, seedlings from a consistent panel of cotton accessions in GWAS were subjected to either control conditions or *V. dahliae* stress. Roots were harvested at 3 and 12 days post‐inoculation (dpi) or under the respective control conditions based on interaction stage between cotton‐*V. dahliae* described in our previous research [38], resulting in a total of 1152 samples from four treatments (labeled as M3, M12 for normal conditions, and D3, D12 for *V. dahliae* stress) for transcriptome sequencing (Figure 4A and Figure S14). Principal component analysis reveals the profound effects of *V. dahliae* infection on gene expression, and a clear distinction between the early and late infection stages was observed (Figure 4B). A total of 59,317 expressed genes (fragments per kilobase of transcript per million mapped reads (FPKM) > 0.1 in at least 5% accessions) were identified among four treatments (Figure 4C). We then analyzed the differentially expressed genes (DEGs) between the control and inoculated groups based on a fold change of expression level > 2 in over 20% of accessions, identifying a total of 20,982 nonredundant *V. dahliae*‐responsive genes (15,796 at the early infection stage and 19,899 at the late infection stage), representing 35.37% of all expressed genes (Figure S15A,B). Furthermore, we categorized these DEGs into three clusters based on expression patterns: *V. dahliae*‐inducible genes (upregulated), *V. dahliae*‐repressed genes (downregulated), and *V. dahliae* responsive‐variable genes (divergent patterns) (Figure S15A,B). In response to biotic threats, chloroplasts act as sources of calcium and ROS signals, transmitting signals to the nucleus and leading to the expression of defense‐related genes [39]. Gene Ontology (GO) analysis revealed that *V. dahliae*‐inducible genes are mainly involved in photosynthesis, transcriptional regulation and ADP binding (predominantly nucleotide‐binding leucine‐rich repeat (NLR) proteins), while *V. dahliae*‐repressed genes and *V. dahliae* responsive‐variable genes are primarily associated with oxidation–reduction processes, response to oxidative stress, transcriptional regulation, systemic acquired resistance and protein phosphorylation (Figures S15C and S16). These findings suggest the crucial role of ROS in the resistance of cotton to *V. dahliae*.

### Dynamic genetic regulation of gene expression in response to *V. dahliae* infection

To investigate the impact of genetic variants on gene expression in cotton seedlings in response to *V. dahliae*, 48,430 genes that showed variations in expression levels exceeding a twofold range between the 5th and 95th percentile levels were retained for further analysis (Figure 4C). We use the same genomic variations data set of GWAS analysis to conduct expression GWAS to investigate the genomic variants that are associated with gene expression. A total of 27,848 cis‐eQTLs were identified for 10,771 genes (cis‐eGenes, genes whose expression was regulated by cis‐eQTLs) (Figure S17A–E and Table S9), only approximately 5% of which had more than one conditionally independent eQTLs, suggesting a relatively simple genetic control of gene expression (Figure 4D). Cis‐eQTLs were enriched in proximity to transcription start site (TSS), 43.26% of which located in the gene body and its flanking 2‐kb regions (Figure S17F,G). In total, 81,790 trans‐eQTLs were identified for 21,420 genes (trans‐eGenes, genes whose expression was regulated by trans‐eQTLs) (Figure S17A–E and Table S9), of which approximately 30% had more than one trans‐eQTLs (Figure 4E). Furthermore, we consistently observed a significant prevalence of interchromosomal associations for trans‐eQTL and target genes between the At‐ and Dt‐subgenomes (Figure S17A–D). This suggests that there is a complex network of genetic interactions between different chromosomes in cotton's response to *V. dahliae* stress. The comparison of cis‐ and trans‐eQTL showed that expression variation (*R* ^2^) of cis‐eQTLs was significantly higher than trans‐eQTLs in all treatments, suggesting that local genetic variants have a greater impact on expression variation compared to distant genetic variants (Figure 4F and Figure S17A–D). The majority of eQTLs exhibited dynamic behavior during both the early and late stages of inoculation (Figure 4G,H), suggesting that gene regulation in response to *V. dahliae* stress is a dynamic and adaptable process. Further analyses of the difference in eGenes during *V. dahliae* infection showed that 41.19% cis‐eGenes and 75.25% trans‐eGenes were related to inoculation (eGenes specifically detected under infection or control) (Figure S17H,I), and GO enrichment analysis revealed that, similar to the functions of *V. dahliae*‐responsive genes, those eGenes were also significantly enriched in oxidation–reduction process, photosynthesis, and regulation of transcription (Figure S17J). This underscores the pivotal role of genomic variants in shaping the expression diversity of defense‐related genes during *V. dahliae* infection.

### Gene modules and regulatory networks involved in coordinated regulation of cotton resistance to *V. dahliae*

To uncover the coordinated regulation of multiple genes influencing cotton resistance to *V. dahliae*, we identified co‐expressed gene modules using Independent Component Analysis (ICA). A total of 608 co‐expressed gene modules were identified (M3: 152, D3: 158, M12: 142, and D12: 156). Among these, 96 modules (M3: 32, D3: 23, M12: 32, and D12: 9) showed significant associations with VW resistance (Table S10). Subsequently, a GWAS was conducted using the expression pattern of each module to identify the regulatory loci for these modules. This analysis revealed one co‐expression module for each treatment condition (named M3‐IC78, D3‐IC7, M12‐IC49, and D12‐IC136) whose associated genomic loci coincided with QTLs. Specifically, QTL1 was associated with all four components, QTL4 was specifically associated with M12‐IC49, and QTL8 was associated with three components excluding M12‐IC49. Additionally, GO enrichment analysis revealed a significant enrichment of ADP binding across all four groups (Figure S18), with genes in this GO term encoding NBS‐LRR genes. Moreover, in the M12 treatment group, ATP binding and protein phosphorylation were significantly enriched, with many genes in these GO terms encoding receptor‐like protein kinases (Figure S18). These findings suggest that three out of ten QTLs (QTL1, QTL4, and QTL8) associated with VW resistance‐related modules may regulate cotton resistance to *V. dahliae* by controlling numerous canonical disease‐resistant genes.

An eQTL hotspot refers to a genomic region where multiple eQTL are concentrated or colocalized that influences the expression of multiple genes. A total of 306 hotspots were identified, regulating 8249 genes with a range from 25 to 286 across four treatment groups. Notably, there were more eQTL hotspots distributed within the Dt subgenome compared to the At subgenome (Dt:At = 197:109). Among these hotspots, 58 and 46 hotspots are completely specific in response to *V. dahliae* at early and late stages of infection, respectively (Figure S19A and Table S11). The genes regulated by the early and late infection stage‐specific hotspots are mainly involved in ADP binding. Moreover, 13 hotspots overlap with 5 QTLs (QTL1, QTL4, QTL5, QTL7, and QTL9) (Table S12). Over half of the genes regulated by hotspot (156) regulate genes exhibit functions similar to *V. dahliae*‐responsive genes, primarily involving oxidation–reduction processes, cell wall remodeling, and ADP binding (Table S13). This highlights the crucial role of trans‐eQTL hotspots in governing the expression of disease resistance‐related genes under *V. dahliae* stress. Based on this, we constructed a regulation network of cotton resistance to *V. dahliae*, consisting of 156 hotspots and 4941 genes (Figure S19B).

### Fine mapping of genes associated with cotton resistance to *V. dahliae*

**Figure 5:**
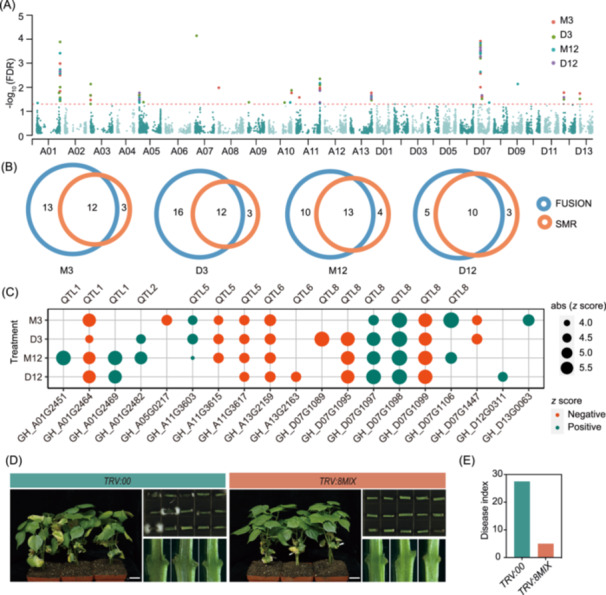
Integrating GWAS and eQTL results prioritize candidate causal genes for cotton resistance to *V. dahliae*. (A) Manhattan plot of the transcriptome‐wide association studies (TWAS) combining data from four treatments. Each point represents an individual gene, genes identified under different treatments are represented in different colors. The red dashed line represents the significance threshold –log_10_ (FDR) = 0.05. (B) Veen diagram showing the number of consistent genes identified by the FUSION and SMR methods. (C) Bubble plot showing the phenotypic effects (TWAS *z* score) of the consistent candidate genes identified in different treatment groups. Red dots represent negative *z* score, dark green dots represent positive *z* score. The size of the dots represents the absolute value of the *z* score. (D) Disease symptoms, fungal recovery assay, and vascular bundle coloration in longitudinal sections of *TRV:00* and *TRV:8MIX* (eight genes simultaneously knocked down) cotton plants. Scale bar, 3 cm. (E) Disease index of *TRV:00* and *TRV:8MIX* cotton plants. eQTL, expression quantitative trait loci; GWAS, genome‐wide association analysis.

To prioritize candidate causal genes for cotton resistance to *V. dahliae*, a TWAS was conducted using the FUSION pipeline by integrating results from GWAS with eQTL data. The analysis identified a total of 47 unique genes (25 genes at M3, 28 genes at D3, 23 genes at M12, and 15 genes at D12), showing significant associations between their expression levels and DI, as determined by a false‐discovery rate (FDR) of *p* < 0.05 (Figures 5A and S20A–D, Table S14). Moreover, summary‐level Mendelian Randomization (SMR) analysis was employed to identify candidate genes whose expression levels were associated with DI due to pleiotropy or causal association. A total of 25 genes passing the HEIDI test (*p*_HEIDI_ > 0.05) were detected, with 15 genes in M3, 15 genes in D3, 17 genes in M12, and 13 genes in D12 (Table S15). Among these genes, 19 out of 25 were consistent with those identified in the TWAS analysis and 15 out of 19 genes were for 5 GWAS QTLs (Figure 5B,C), suggesting that the cis‐regulated expression level for these genes mediates the association between genetic variants and DI. Notably, 6 causal candidate gens were founded in QTL8, and 13 out of 19 genes were also regulated by trans‐eQTL hotspots, with *GH_D07G1095* in QTL8 being the only one gene regulated by an eQTL hotspot (Hot102) overlapping with GWAS loci (QTL5) (Table S12). In summary, we prioritize these 15 genes as promising candidate causal genes for cotton resistance to *V. dahliae* (Figure 5C). *Z*‐scores from TWAS results and correlation analysis between gene expression levels and DI revealed that the expression levels of eight genes were negatively associated with cotton resistance to *V. dahliae* (Figure 5C and Table S16). To confirm the role of these eight genes in cotton resistance to *V. dahliae*, individual knockdowns and simultaneous knockdowns of these genes were conducted. Significant improvement in VW resistance was observed in the *TRV:negx* seedlings, particularly in the *TRV:8MIX* group, as evidenced by reduced chlorosis, lower disease index, diminished brown coloration of vascular tissues, and decreased fungal biomass (Figures 5D,E and S21). The results indicate the potential benefits of targeting these eight genes for cotton disease resistance breeding.

### *GhARM* modulates cotton's VW resistance by regulating ROS homeostasis

**Figure 6:**
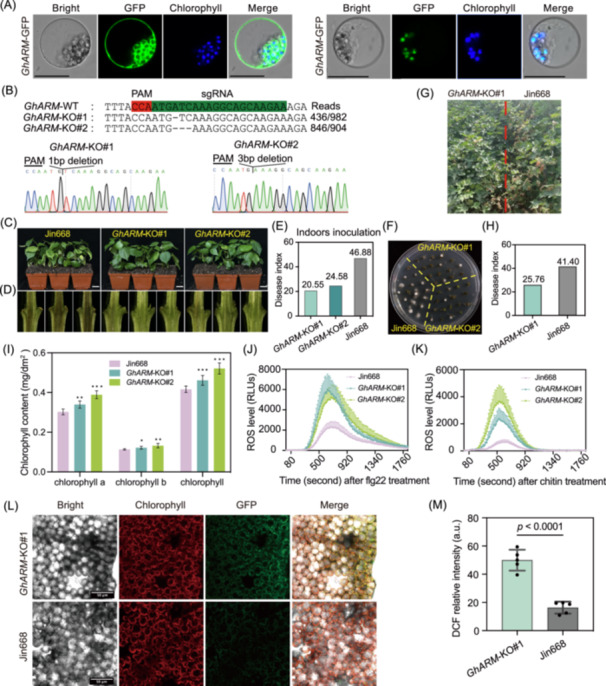
*GhARM* modulates cotton's VW resistance by regulating reactive oxygen species (ROS) burst. (A) Subcellular location of *GhARM* in cotton protoplast. Scale bar, 20 μm. (B) Mutation type of *GhARM* knockout lines. Two representative knockout lines, *GhARM*‐KO#1 and *GhARM*‐KO#2 were generated in the Jin668 genetic background. Disease symptoms (C), vascular bundle coloration in longitudinal sections (D), disease index (E), and fungal recovery assay (F) of wild‐type and *GhARM* knockout cotton plants at 12 days pos‐tinoculation with v991. Scale bar, 3 cm. (G) Comparison of resistance to *V. dahliae* between Jin668 and *GhARM* knockout cotton plants in the field. (H) Disease index statistic of Jin668 and *GhARM* knockout plants in the field. (I) Contents of chlorophyll a, chlorophyll b, and total chlorophyll in leaves of the Jin668 and *GhARM* knockout cotton plants. Data are displayed as mean values ± SEM (*n* = 6 biological replicates). Various asterisks denote significant differences assessed using one‐way ANOVA, by comparing the mean of *GhARM* knockout lines with that of wild‐type (*p < 0.05, **p < 0.01, ***p < 0.001). Flg22 (J) and chitin‐induced (K) ROS production in leaves of the Jin668 and *GhARM* knockout cotton plants. Error bars represent ± SEM. (L) Confocal microscopic observation of H_2_DCFDA probed H_2_O_2_ accumulation in leaf mesophyll cells of wild‐type Jin668 and *GhARM* knockout seedlings at 3 days post‐inoculation with v991. (M) Quantification of H_2_O_2_ fluorescence intensity in leaf mesophyll cells. Data are represented as mean values ± SD for six independent leaves per line. The asterisks indicate statistically significant differences between wild‐type and *GhARM* knockout lines; Student's *t*‐test; *n* = 5 biological replicates. VW, Verticillium wilt.

ROS homeostasis plays an important role in cotton‐*V. dahliae* interaction [38]. Among the 8 genes that negatively regulate cotton resistance to *V. dahliae*, *GhARM* (*GH_D07G1095*) from QTL8 encodes a protein of unknown function with four tandem arrangement of Armadillo (ARM)‐repeat domains. Subcellular localization analysis revealed that *GhARM* is localized in the plasma membrane, cytoplasm, and chloroplasts (Figure 6A). Two *GhARM* knockout lines (*GhARM*‐KO#1 and *GhARM*‐KO#2) were generated (Figure 6B), and the mutants showed significantly reduced disease symptoms, decreased disease index, and diminished vascular browning at 12 dpi compared to the wild‐type Jin668 (Figure 6C−E). Fungi recovery assay demonstrated a significant reduction in pathogen accumulation in the stem of the *GhARM*‐KO#1 and *GhARM*‐KO#2 plants (Figure 6F). The resistance of *GhARM* knockout mutants to *V. dahliae* was evaluated in disease nursery, and the results also indicated a substantial enhancement in tolerance compared to the wild‐type Jin668 with normal agronomic traits, including plant height, length of the first node of the fruit branch, number of leaf branch, and lint percentage (Figures 6G,H and S22). Transcriptomic analysis revealed significant enrichment of chlorophyll biosynthetic processes, with upregulated expression of relevant genes in the *GhARM* mutant (Table S17 and Figure S23). Measurements of chlorophyll content also indicated a higher chlorophyll a/b content in the *GhARM* mutant plants than that in the wild‐type (Figure 6I). Additionally, DEGs were also mainly enriched in the categories of oxidation–reduction processes, response to oxidative stress, and plant immune, with a notable decrease in the expression of peroxidase in the *GhARM* knockout mutants (Table S17 and Figure S23). To validate that *GhARM* may regulate cotton resistance to *V. dahliae* by modulating the ROS homeostasis, we measured ROS production in cotton leaves treated with pathogen‐associated molecular patterns (PAMPs) flg22 and chitin. The results indicated significantly increased ROS bursts in *GhARM*‐KO#1 and *GhARM*‐KO#2 (Figure 6J,K). Furthermore, 2',7'‐dichlorodihydrofluorescein diacetate (H_2_DCFDA) was used to probe chloroplast‐mediated ROS (cROS) in cotton leaves. A significant increase in green fluorescence signals was observed in the *GhARM* mutant (Figure 6L,M). Overall, the *GhARM* gene probably modulates cotton's VW resistance by regulating cROS homeostasis.

Analysis of natural variations across *GhARM* among 290 cotton accessions in this study identified two main haplotypes (HAP1 and HAP2), with accessions carrying HAP2 exhibited lower gene expression and DI compared to those with HAP1, indicating enhanced resistance of *GhARM* ^*HAP2*^ (Figures S24A,B and S25). To explore the evolution of *GhARM* for VW resistance, genomic variations in the upstream 2 kb region and the sequences of *GhARM* were analyzed in 3516 upland cotton accessions [40], including 237 wild types, 138 landraces, and 3141 cultivars. A total of 10 SNPs and 2 Indels were detected, resulting in the identification of 7 major haplotypes (HAP1–HAP7) for *GhARM* (Figure S24C,D). The distribution of these haplotypes varied significantly among wild types, landraces, and cultivars. Wild types exhibited a diverse range of haplotypes, predominantly HAP2 and HAP3. Compared to wild types, the frequency of HAP2 increased in landraces, while there was no notable change in its proportion in modern cultivars, indicating substantial room for improvement (Figure S24E,F). Further analysis estimated the nucleotide diversity (π) and Tajima's D across the 300 kb genomic region flanking the *GhARM* gene among wild cotton, landraces, and cultivars, revealing low diversity in both in type (Figure S24G), indicating evolutionary constraints on *GhARM*. Notably, wild types exhibited higher nucleotide diversity than landraces and cultivars (Figure S24G). Tajima's D calculations revealed that most regions in landraces and cultivars showed negative Tajima's D values, while values in wild cotton were predominantly positive (Figure S24H), indicating a history of selection pressure surrounding the genomic region of *GhARM* in cotton. Collectively, these findings imply that *GhARM* underwent artificial selection during the domestication and improvement of cotton.

## DISCUSSION

### The excavation and pyramiding of elite alleles comprehensively enhance the VW resistance of cotton

The accumulation and development of *V. dahliae* in the disease field caused by the recirculation of straw and continuous monocropping, together with the complex composition of *V. dahliae* strains and soil microbiota community and the scarcity of resistance gene resources in upland cotton, collectively exacerbate the challenges associated with the management of VW in cotton crops. Despite the reporting of more than 200 VW QTLs and genes with minor effect [8, 9, 10, 41, 42], the lack of accurate QTL mapping and verification of functional genes has hindered the effective use of markers and genes for breeding cotton with VW resistance. Pyramid breeding, similar to improving oil content in *Brassica napus* and fiber quality in cotton [20, 28], is an efficient strategy for improving complex traits such as VW resistance in cotton. Isolates of the pathogen originating from disease fields in Xinjiang show significant differences in growth and continuous variations in pathogenicity [43]. Similar results were observed with VW in mint, olive, and potato fields [44]. Stable effects of 10 QTLs are observed in different environments. Of these, *qVWR.A10.3* (QTL4) and *qVWR.D07.1* (QTL8) show similarities with previously reported QTLs [10, 45], while the remaining 8 QTLs are newly identified. Pyramiding all 10 Lsnp^R^s proves to be an effective strategy for improving cotton resistance, whether to diseases in the field or inoculations with *V. dahliae* strains. The presence of 10 Lsnp^R^s in ZZM2 provides an explanation for the stable resistance to *V. dahliae*. In particular, the specific geographical distribution of pathogen populations and different agricultural practices can significantly influence the resistance performance of cotton, which ultimately affects the selection of varieties. The fixed Lsnp1^R^ and Lsnp4^R^ in China are elite loci selected under disease and show the most striking effects and high stability. The *Fov7*‐resistant allele, which serves as the major gene for cotton's Fusarium wilt resistance, was selected in the FW‐inoculated field and made a great contribution to the stable Chinese cotton production [31]. Notably, the use of Lsnp1^R^, Lsnp4^R^, Lsnp5^R^, Lsnp8^R^, and Lsnp9^R^ has increased significantly since the 1990s, possibly due to the improvement in disease resistance improvement as several major outbreaks of VW occurred in the 1990s and 2000s. We hypothesize that MF and LF Lsnp^R^ have contributed significantly to the improvement of VW resistance in breeding programs. The fluctuation in the frequency of 10 Lsnp^R^s in varieties developed in different eras is complex, implying that 10 Lsnp^R^s were not subjected to explicit directional selection, possibly due to the trade‐off between disease resistance and yield, where improving yield traits may come at the cost of resistance. In future breeding for disease resistance, prediction using the MDIC can be used to selectively enhance the missing Lsnp^R^, especially Lsnp1^R^, Lsnp4^R^, Lsnp5^R^, Lsnp8^R^, and Lsnp9^R^, to precisely improve resistance to *V. dahliae*.

The homogeneous genetic background of upland cotton is a limitation for improving disease resistance. *Fhb7*, derived from the poorly related wild species *Th. elongatum*, provides durable resistance to Fusarium head blight in wheat [46]. In the history of cotton breeding, efforts have been made to improve the yield, quality, and especially the resistance of upland cotton through interspecific hybridization. In addition, resistance genes against *V. dahliae* have been found in the genomes of *G. arboretum* [12]. The breeding process of ZZM2 involved hybridization with the VW‐resistant variety *G. barbadense* ‘duomaozao’ [47], suggesting that exploration and utilization of resistance gene reservoirs from other cotton species is a strategy to overcome the limitations of the genetic background of upland cotton.

### eQTL plays an important role in shaping the expression diversity of defense‐related genes during biotic stress

GWAS has proven to be a powerful method to study the genetic basis of complex traits in diverse crops [48]. Integration of GWAS and eQTL analyses stands as a powerful approach to further understanding the causal genetic variants underlying complex traits through regulatory effects on the expression of target genes in post‐GWAS era [49]. While a few studies have focused on genetic variation regulating gene expression under abiotic stress in plants, such as drought stress [15, 16] and heat stress [18], to our knowledge, this is the first study delving into the genetic variation governing gene expression under biotic stress. In this study, a total of 109,638 eQTLs associated with 25,187 genes were identified, providing a valuable resource for post‐GWAS research on cotton resistance to *V. dahliae*. The involvement of DEGs under *V. dahliae* stress and genes regulated by eQTLs exhibits similar roles, illustrating the significant impact of genomic diversity on the expression of disease resistance‐related genes. This may be related to the breeding history of cotton in China, where VW has long been a primary threat to cotton production. Breeders have emphasized breeding varieties with VW resistance, resulting in a close relationship between genomic diversity and the expression of disease‐resistance genes in diverse cotton varieties. By integrating GWAS and eQTL data, we clarified the genetic basis and regulatory mechanisms of VW resistance in cotton, shedding light not only on this crop but also on broader implications for plants in response to disease stress.

### ROS homeostasis plays a vital role in defense signaling

As research advances, a growing number of susceptibility genes in plants, such as *Mlo* in wheat [50], *SWEET* genes in rice [51], and RLCK *TaPsIPK1* in wheat [52], have been identified. Disruption of susceptibility genes through techniques like gene editing is considered an attractive strategy for crop resistance breeding [53]. Upland cotton has a narrow genetic background, limited disease‐resistant germplasm, and overall poor disease resistance, leading to weak effects of disease‐resistant genes and making them challenging to utilize in breeding. Therefore, exploring susceptibility genes in cotton and utilizing genome editing for gene editing represent a more effective strategy for breeding VW‐resistant cultivars compared to relying solely on resistance genes. In this study, we confirmed the function of eight negative regulatory causal genes and discovered that simultaneous knockdown of all eight genes further strengthens cotton resistance to *V. dahliae*. However, the potential synergistic regulatory effects of these eight negative regulatory genes and their underlying mechanisms require further exploration. After knocking out one of the negative regulatory genes (*GhARM*) using gene editing, both indoor inoculation and field disease resistance assessments showed a significant enhancement in resistance to *V. dahliae*. Future research could employ a multi‐gene editing system to concurrently target these negative regulatory genes and develop Cas9‐free plants.

The *GhARM* gene is located in the chloroplast, suggesting its potential role in negatively regulating cotton resistance to *V. dahliae* by modulating chloroplast‐mediated ROS homeostasis. As research progresses, chloroplasts, crucial for photosynthesis and plant growth, are recognized for their vital role in plant immunity [39]. Chloroplasts are the primary organelles responsible for ROS production under stress conditions. However, the maintenance of chloroplastic ROS homeostasis during *V. dahliae* infection remains largely unknown. In this study, increased chloroplastic ROS levels were observed in cotton plants after knocking out *GhARM* in response to *V. dahliae*, potentially contributing to enhanced resistance against VW. Our previous research revealed that *V. dahliae* induces ROS production to facilitate infection [38], indicating a discrepancy between chloroplastic ROS and total ROS involvement in cotton resistance against *V. dahliae*. Given that chloroplasts play an important role in plant growth and immune regulation, they are vulnerable to pathogen attacks during plant‐pathogen interactions [54, 55]. How *GhARM* regulates chloroplast ROS homeostasis needs further study.

## CONCLUSION

In summary, our study elucidates the genetic basis and regulatory landscape of cotton resistance to *V. dahliae*, as well as genetic foundation of advancements in cotton VW‐resistance breeding, offering crucial insights into the role of pyramiding QTLs in complex agronomic traits. Utilizing 10 QTLs identified across various environments, we established an effective genomic selection system for VW‐resistant breeding in cotton. The predictive power of these 10 QTLs in assessing cotton resistance to *V. dahliae* was validated using an F_2:3_ population, providing valuable strategies for breeding superior cultivars via genomic selection in cotton breeding. Integration of genomic, population transcriptomic, and functional genomes emphasized the significant role of ROS in cotton's VW resistance. The findings offer valuable genetic resources for disease‐resistant breeding in cotton and significant insights into understanding the genetic basis and regulatory mechanisms of VW resistance in cotton and other crops.

## METHODS

### Plant materials and field assays

The upland cotton population used in this study consists of 290 cotton accessions derived from China and was previously re‐sequenced by our laboratory [31, 56]. These accessions were planted in natural VW nurseries located in Manasi (M), Korla (K), and Kuqa (C) regions of Xinjiang, China. Following disease onset, DI was collected for one, two, and 2 years in the respective locations, resulting in five independent environments (18M, 18K, 18C, 19K, and 20C). With the exception of a single replication in 20C, all other years and locations had two replications, resulting in a total of nine sets of raw DI (18M‐1, 18M‐2, 18K‐1,18K‐2, 19K‐1, 19K‐2, 18C‐1, 18C‐2, and 20C‐1). The best linear unbiased estimates (BLUE) were used for replicated locations and replications (M‐b, 18K‐b, 18C‐b, 19K‐b, K‐b, and C‐b). Moreover, by using BLUE for the all nine raw DI derived a comprehensive DI (All‐b). Additionally, each accession which had replication in each year and location selected the replication with the higher DI as the accurate DI. This resulted in merged DI for four sets (18M‐3, 18K‐3, 18C‐3, and 19K‐3), totaling 20 sets of DI (Figure 1A).

### Genomic variant calling

The raw re‐sequenced data generated in our previous study was subjected to trimming and filtering using the fastp (v0.20.0) software [57]. SNP calling was performed using the standard pipeline of Sentieon (v201911) [58]. Specifically, we utilized the Sentieon tools to align the trimmed reads to the TM‐1 reference genome [59] using BWA (v0.7.17) [60]. Further, we removed duplicated reads and poorly mapped reads using SAMtools (v1.9) [61]. The Sentieon Haplotyper algorithm was then employed for SNP calling, with the following parameters: ‐‐algo Haplotyper ‐‐emit_conf=10 ‐‐call_conf=10 ‐‐emit_mode gvcf. This step generated a GVCF file for each sample, containing information about the detected variations. The variations from all samples were subsequently merged into an integrated VCF file using the GVCFtyper algorithm. Finally, the integrated VCF file was filtered using VCFtools (v0.1.16) [62], keeping only SNPs with a depth (DP) greater than 5, missing rate (missing) less than 50, and a minimum allele frequency above 0.05.

### Genome‐wide association studies

A total of 2,152,153 high‐quality variants were extracted from 290 accessions. GWAS analysis was performed using the GEMMA (v0.98.1) software [63], applying a mixed linear model to perform association analysis between genomic variants and DI of 20 group phenotype. The population structure was inferred using the STRUCTURE (v2.3.4) software [64], employing a subset of 100,000 SNPs. The kinship was assessed using all SNPs via GEMMA. The significance threshold for GWAS was set at an empirical threshold of 1E‐05 and Bonferroni correction threshold of 4.65E‐05 (1/*n*, *n* is the total number of SNPs). Distinct loci within the same linkage disequilibrium (LD) (*r* ^2^ ≥ 0.5) were merged into a single QTL.

### Genomic prediction for DI to *V. dahliae* using ridge regression

The glmnet R package (v4.0‐2), utilizing a linear regression with penalty to reduce overfitting, was employed to fit model and conduct phenotype prediction [65]. The 10 stable QTLs were designated as the predictor variables and 20 DI sets were considered the target variable. Ridge regression was used to fit the model through the cv. glmnet() function, setting alpha = 0 to control the regularization. Module tuning was done by performing cross‐validation to select the optimal model hyperparameters. The value of λ (regularization parameter) that corresponds to the minimum cross‐validated minimum mean squared error was selected to perform phenotype prediction using the function predict() in glmnet.

### Population transcriptome sequencing

We cultivated the 290 cotton accessions in the greenhouse at Huazhong Agricultural University using hydroponic cultivation method, as illustrated in Figure 4A. The materials were placed in plastic trays measuring 42 cm × 30 cm, filled with Hoagland nutrient solution (NS10205; Coolaber), and secured with sponges. Ten seedlings were prepared for each material. The v991 strain was used for inoculation and incubated in Czapek solution (MM1020‐C; Coolaber). After 15 days of seedling growth, we performed separate inoculation treatments with a spore concentration of 1 × 10^7^ spores/mL and Mock treatments (using a solution with the same concentration of Czapek as the inoculation treatments). Root tissues and approximately 1 cm of hypocotyl of five seedlings were collected after 3 and 12 days of treatment, respectively, except for samples J17 and J36, which failed to be collected. As a result, each treatment group comprised 288 materials. In total, we obtained 1152 samples for RNA extraction and sequencing. RNA extraction utilized the RNAprep Pure Plant Plus Kit (Polysaccharides and Polyphenolics‐rich) (DP441; TIANGEN). Library construction and sequencing were performed by Personal Biotechnology Co., Ltd. using the VAHTS Universal V6 RNA‐seq Library Prep Kit (NRM604‐02; Vazyme) and NovaSeq. 6000 platform (pair‐end 150 bp reads), respectively.

### eQTL mapping analysis

Genes with an FPKM value greater than 0.1 in materials exceeding 5% are defined as expressed genes. Furthermore, genes with expression variation between the 5th and 95th percentiles that changed less than a two‐fold change are filtered out. After this filtering process, each of the four groups (M3, D3, M12, and D12) retained a specific number of genes (42,173, 43,014, 46,703, and 41,644) for eQTL analysis. Considering batch effects and experimental confounders, we utilized the probabilistic estimation of expression residuals (v1.0) method to estimate confounding factors [66]. Incorporating these factors as covariates enhances the power and interpretability of gene expression analyses. Principal components based on genotype were computed using Plink to infer population structure. Subsequently, FPKM of each gene was quantile‐quantile normalized using the R package “qqnorm,” and the linear regression model from the TensorQTL software (v1.0.8) was employed for eQTL mapping [67]. The top 20 hidden and confounding factors, as well as the first five PCA, were considered as covariates.

eQTLs are classified as either cis‐eQTLs, if they are located within 1 Mb of the gene, or as trans‐eQTLs if they are located more than 1 Mb away or on different chromosomes. For cis‐eQTLs, we identified conditionally independent cis‐eQTLs using a combination of permutation and stepwise regression approach. The cis window flanking each gene's TSS was set to 1 Mb, with parameters set as ‐‐cis ‐‐window 1,000,000 ‐‐qvalue_lambda 0.05. This method utilizes the Beta distribution to calculate empirical *p*‐values, which are then used to calculate the genome‐wide FDR. SNPs with a *q*‐value less than 0.05 were deemed to be significantly associated with gene expression and classified as cis‐eQTLs. Genes with significantly associated cis‐eQTLs were considered as cis‐eGenes.

For trans‐QTL mapping, default parameters were used, then cis‐associations within 1 Mb of the gene's TSS were removed. The significance threshold for trans‐eQTLs was calculated using the Genetic Type 1 Error Calculator (GEC) (v1.0) to determine the effective number of independent SNPs [68]. According to the GEC calculation, the suggestive *p*‐value was 2.45E‐06 (1/364,248). Given the abundance of trans‐eQTLs, for each gene, SNPs within a continuous 10 kb region were grouped into one cluster, and only the cluster with at least three significant SNPs was considered as an eQTL. The most significant SNP was designated as the representative eQTL. Additionally, eQTLs for each expression trait within the same linkage disequilibrium (LD) (*R* ^2^ > 0.1) were merged into one eQTL. Trans‐eQTL hotspots for each treatment was identified using permutation‐based method. Trans‐eQTLs were randomly assigned to genomic regions, and a sliding window analysis was conducted with 1‐Mb window size and a 100‐kb step size to scan the number of eQTLs. The threshold number of eQTLs was determined based on the distribution of the maximum number of eQTLs from 1000 permutations with a significance threshold of *p* = 0.01.

### TWAS for cotton resistance to *V. dahliae*

We utilized FUSION package [69] to conduct TWAS to identify genes whose cis‐regulated expression is associated with cotton resistance against *V. dahliae*. Following the official workflow, we initially construct standard binary PLINK format files for each cis‐eGene using PLINK1.9. This involved utilizing the normalized expression values of each cis‐eGene as phenotypes and selecting the SNPs located within 1 Mb upstream and downstream of each cis‐eGene as genotypes. We then employed the script FUSION compute_weights.R in FUSION to calculate expression weights and performance statistics for each gene, utilizing five models (BLUP, BSLMM, LASSO, Elastic Net, and top SNPs). Finally, we performed a typical TWAS analysis using the script FUSION.assoc_test.R, incorporating the previously determined weights and summary association statistics obtained from GWAS results as the input. Genes demonstrating an FDR‐adjusted *p*‐value below 0.05 were deemed significantly associated with *V. dahliae* resistance.

### SMR for colocalization analysis

The SMR & HEIDI methods implemented in the SMR software (v1.03) [70] were used to assess whether the effect size of SNP on *V. dahliae* resistance was influenced by gene expression. Cis‐eQTL summary data for each treatment were generated by SMR in binary format (BESD), and SMR analysis was conducted to assess the association between gene expression and *V. dahliae* resistance by integrating the cis‐eQTL data with summary‐level data obtained from a GWAS of 20 DI sets. Genes with a *p*‐value lower than 1/*n* (where *n* represents the number of cis‐eGenes) were considered to have a significant association with *V. dahliae* resistance.

### Coexpression module construction by ICA

To construct a coexpression module, gene expression matrices for different samples were standardized using the scale function in the R package. Subsequently, the matrix decomposition was performed using the runICA function in the R package picaplot (v0.99.7), resulting in the matrix product *X* = *S × A*. The parameters were set as var_cutoff = 80, max_iter = 10, n_runs = 15 (https://github.com/jinhyunju/picaplot). The matrix *S* consists of genes as rows and components as columns, enabling the assignment of genes to specific components, while matrix A is composed of components as rows and different samples as columns, reflecting the expression patterns of each component. Association analysis between components and VW resistance was conducted using EMMAX software (v20120205).

### Vector construction

For knockdown of eight candidate causal genes through VIGS, ~300–500 bp PCR fragments of target gene were cloned from cDNA and inserted into the BamHI and KpnІ restriction sites of the TRV vectors using ClonExpress II One Step Cloning Kit (C112, Vazyme). For knockout of *GhARM*, the sgRNA was cloned into CRISPR/Cas9 binary vector pRGEB32, under the control of *GhU6* promoter. For the transient expression assay in protoplasts, the coding sequence of *GhARM* was amplified from cDNA and cloned into pHBT vectors, driven by CaMV 35S promoter with a GFP tag at C‐terminus. The related primers are listed in Table S18.

### Pathogen infection assays

We inoculated cotton plants with the defoliating strain v991 of *V. dahliae*. The strain stored at −80°C was initially reactivated by culturing on potato dextrose agar plates, then transferred to sterilized Czapek‐Dox medium and incubated at 25°C on a shaker for 3–4 days. Following filtration of the culture through gauze, the spore concentration was adjusted to 2 × 10^5^ spores/mL. Inoculation was achieved using the root‐dip method, with cotton roots immersed in the spore suspension for 2 min, followed by transplanting the plants to into a blended soil mixture of nursery substrate and vermiculite. Disease symptoms were observed 12 dpi, and disease index was calculated. For RNA sequencing of Jin668 and *GhARM* knockout mutants, 2‐week‐old seedlings were inoculated with v991, and roots were collected at time points of 0 and 3 dpi.

### Measurement of chlorophyll content

For the measurement of chlorophyll content, six leaf discs of each genotype were submerged in 1 mL of 95% (v/v) ethanol in a 2 mL tube. The tubes were sealed with parafilm, kept in the dark at 4°C for 12 h for extraction. The chlorophyll content was determined using EnSpire Multimode Plate Reader (PerkinElmer) at wavelengths of 645 and 663 nm to measure the absorbance of the chlorophyll extracts. Chlorophyll content was then calculated using the formulas: chlorophyll *a* = (12.72*A*_663_ − 2.59*A*_645_) × *V*/*W* × 1000, chlorophyll *b* = (22.88*A*_645_ − 4.67*A*_663_) × *V*/*W* × 1000, total chlorophyll content = (20.29*A*_645_ + 8.05*A*_663_) × *V*/*W* × 1000. Here, *V* represents the volume of the extraction solution (mL), and *W* denotes the leaf area (dm^2^).

### ROS detection

For the measurement of ROS using a luminol‐based assay, 3‐week‐old cotton leaves from wild‐type and *GhARM* knockout plants were excised into 5 mm diameter leaf discs. The leaf discs were incubated overnight at room temperature in a 96‐well plate with sterile water to eliminate damage effects. The following day, the leaf discs were treated with a solution containing 50 μM luminol (Sigma‐Aldrich), 20 μg/mL peroxidase from horseradish (Sigma‐Aldrich), 100 nM flg22, or 100 μg/mL chitin. Luminescence was recorded using an EnSpire Multimode Plate Reader (PerkinElmer) for approximately 30 min. Detection of ROS production in cotton leaf mesophyll cells was achieved using H_2_DCFDA under confocal microscopy. Following the method described previously [38], leaf discs were immersed in 25 μM H_2_DCFDA and then incubated in darkness for 30 min. After the incubation period, the leaf discs were rinsed three times with deionized water. Images were captured using a Leica SP8 microscope with a 488 nm excitation wavelength and 500–600 nm emission range, while chlorophyll autofluorescence was observed at 640–735 nm.

## AUTHOR CONTRIBUTIONS

**Xiaojun Zhang**: Methodology; software; data curation; investigation; validation; formal analysis; visualization; writing—original draft. **Shiming Liu**: Methodology; software; investigation; validation; formal analysis; visualization; writing—original draft. **Peng Wu**: Software; data curation; investigation; validation; formal analysis. **Wanying Xu**: Data curation; investigation; validation; formal analysis. **Dingyi Yang**: Investigation; validation; formal analysis. **Yuqing Ming**: Investigation; validation. **Shenghua Xiao**: Methodology; investigation. **Weiran Wang**: Investigation; resources. **Jun Ma**: Investigation; resources. **Xinhui Nie**: Investigation; resources. **Zhan Gao**: Investigation; validation. **Junyuan Lv**: Investigation; validation. **Fei Wu**: Investigation; validation. **Zhaoguang Yang**: Investigation; validation. **Baoxin Zheng**: Investigation; visualization. **Ping Du**: Investigation; formal analysis. **Jiangmei Wang**: Investigation; formal analysis. **Hao Ding**: Investigation. **Jie Kong**: Investigation; resources. **Alifu Aierxi**: Investigation; resources. **Yu Yu**: Investigation; resources. **Wei Gao**: Methodology; supervision; writing—review and editing; resources. **Zhongxu Lin**: Writing—review and editing; resources. **Chunyuan You**: Investigation; resources. **Keith Lindsey**: Supervision; writing—review and editing. **Nataša Štajner**: Writing—review and editing; supervision. **Maojun Wang**: Supervision; writing—review and editing. **Jiahe Wu**: Writing—review and editing; supervision. **Shuangxia Jin**: Writing—review and editing; supervision; conceptualization. **Xianlong Zhang**: Funding acquisition; conceptualization; writing—review and editing; project administration; supervision. **Longfu Zhu**: Funding acquisition; writing—review and editing; supervision; project administration; conceptualization.

## CONFLICT OF INTEREST STATEMENT

The authors declare no conflicts of interest.

## ETHICS STATEMENT

No animals or humans were involved in this study.

## Supporting information


**Figure S1:** Cotton accessions represent the occurrence phenotypes in fields affected by *V. dahliae*.
**Figure S2:** Histogram of the frequency distribution of DI of 290 upland cotton accessions in five independent environments.
**Figure S3:** Correlation between DI in different environments and the BLUE adjusted DI.
**Figure S4:** Cluster analysis of DI of 290 upland cotton accessions across five environments.
**Figure S5:** Manhattan plot of 20 DI sets in 290 accessions.
**Figure S6:** Collected information on the breeding era and geographic distribution of the CDCs.
**Figure S7:** Comparison of DI among 290 upland cotton accessions from the four major cotton‐growing regions.
**Figure S8:** Pyramiding analysis of 10 Lsnp^R^s among the upland cotton accessions.
**Figure S9:** The DI distribution of simulated varieties carrying different numbers of Lsnp^R^.
**Figure S10:** Field resistance phenotypes of the parents used for constructing the artificial population.
**Figure S11:** Density plot of high‐throughput sequencing genotypes in 272 F_2_ individuals at 5 Lsnps.
**Figure S12:** Pyramiding analysis of Lsnp^R^s among F_2:3_ lines with exclusion of the six extreme F_2:3_ lines.
**Figure S13:** The effect of enhancing VW resistance in existing cotton materials after transformation from Lsnp^S^ to Lsnp^R^.
**Figure S14:** Sampling design of the population transcriptome at 3 and 12 days post‐inoculation with *V. dahliae*.
**Figure S15:** Expression pattern clustering and GO enrichment analysis of differentially expressed genes (DEGs) in response to *V. dahliae* infection.
**Figure S16:** GO enrichment analysis of differentially expressed genes (DEGs) in response to *V. dahliae* infection involved in molecular function and cell cellular.
**Figure S17:** Distribution and annotation of eQTLs and functions of eGenes involved.
**Figure S18:** GO enrichment analysis of genes in co‐expressed gene modules associated with QTLs.
**Figure S19:** Distribution of trans‐eQTL hotspots and gene regulatory networks correlated with *V. dahliae* resistance.
**Figure S20:** Individual Manhattan plots of the transcriptome‐wide association study analysis for four treatments.
**Figure S21:** The individual knockdown of eight negatively regulated genes resulted in improved cotton resistance to *V. dahliae*.
**Figure S22:** Field evaluation for agronomic trait of Jin668 and *GhARM* knockout cotton plants.
**Figure S23:** Heatmap for the expression of genes involved in chlorophyll biosynthetic process and response to oxidative stress.
**Figure S24:** Natural variation and selection footprint analysis of *GhARM*.
**Figure S25:** Comparison of DI for all 20 DI sets among individuals with the two main haplotypes of *GhARM* in 290 cotton accessions.

**Table S1:** Summary of 290 upland cotton accessions.
**Table S2:** 20 sets of disease index of 290 upland cotton accessions.
**Table S3:** List of 22 significant loci associated with cotton resistance to *V. dahliae*.
**Table S4:** Source information for cultivars in Chinese variety pool.
**Table S5:** Genotypes of 290 upland cotton accessions at 10 Lsnps.
**Table S6:** Genotypes and pedigrees for the F_2:3_ lines at 10 Lsnps.
**Table S7:** The phenotypic statistics for 19 F_2:3_ lines after inoculation.
**Table S8:** Summary of the comparative analysis between materials with S genotype and R genotype at 10 Lsnps.
**Table S9:** Summary of eQTL data during *V. dahliae* infection.
**Table S10:** Summary of Co‐expressed gene modules associated with cotton resistance to *V. dahliae*.
**Table S11:** Summary of hotspot and its regulated eGenes.
**Table S12:** Summary of function hotspot and its regulated eGenes.
**Table S13:** Gene Ontology enrichment analysis of genes regulated by hotpsots.
**Table S14:** Summary of genes identified through TWAS.
**Table S15:** Summary of genes identified through SMR analysis.
**Table S16:** Information of eight causal genes that negatively regulate cotton's VW resistance.
**Table S17:** Gene Ontology enrichment analysis of differentially expressed genes between wild type and *GhARM* knockout plants.
**Table S18:** Primer sequences used in the paper.

## Data Availability

The raw sequencing data produced in this study have been archived in the National Center for Biotechnology Information database under the accession number PRJNA1195373 for the RNA‐seq data (https://www.ncbi.nlm.nih.gov/bioproject/?term=PRJNA1195373). The data and scripts used are saved in GitHub https://github.com/smliu-lshm/cotton_VW_QTL. Supplementary materials (figures, tables, graphical abstract, slides, videos, Chinese translated version, and update materials) may be found in the online DOI or iMeta Science http://www.imeta.science/.
