# Supplementary material for: A panoramic view of cotton resistance to Verticillium dahliae: From genetic architectures to precision genomic selection
Source: Imeta. 2025 Apr 11;4(3):e70029. doi: 10.1002/imt2.70029 (PMC12130556; doi:10.1002/imt2.70029)
Supplement: Supplementary file 1 — Figure S1: Cotton accessions represent the occurrence phenotypes in fields affected by V. dahliae. Figure S2: Histogram of the frequency distribution of DI of 290 upland cotton accessions in five independent environments. Figure S3: Correlation between DI in different environments and the BLUE adjusted DI. Figure S4: Cluster analysis of DI of 290 upland cotton accessions across five environments. Figure S5: Manhattan plot of 20 DI sets in 290 accessions. Figure S6: Collected information on the breeding era and geographic distribution of the CDCs. Figure S7: Comparison of DI among 290 upland cotton accessions from the four major cotton‐growing regions. Figure S8: Pyramiding analysis of 10 LsnpRs among the upland cotton accessions. Figure S9: The DI distribution of simulated varieties carrying different numbers of LsnpR. Figure S10: Field resistance phenotypes of the parents used for constructing the artificial population. Figure S11: Density plot of high‐throughput sequencing genotypes in 272 F2 individuals at 5 Lsnps. Figure S12: Pyramiding analysis of LsnpRs among F2:3 lines with exclusion of the six extreme F2:3 lines. Figure S13: The effect of enhancing VW resistance in existing cotton materials after transformation from LsnpS to LsnpR. Figure S14: Sampling design of the population transcriptome at 3 and 12 days post‐inoculation with V. dahliae. Figure S15: Expression pattern clustering and GO enrichment analysis of differentially expressed genes (DEGs) in response to V. dahliae infection. Figure S16: GO enrichment analysis of differentially expressed genes (DEGs) in response to V. dahliae infection involved in molecular function and cell cellular. Figure S17: Distribution and annotation of eQTLs and functions of eGenes involved. Figure S18: GO enrichment analysis of genes in co‐expressed gene modules associated with QTLs. Figure S19: Distribution of trans‐eQTL hotspots and gene regulatory networks correlated with V. dahliae resistance. Figure S20: Individual Man [file IMT2-4-e70029-s002.docx]

**Supporting information to**

**A panoramic view of cotton resistance to *Verticillium* *dahliae*: from genetic architectures to precision** **genomic selection**

**Running title**: Cotton resistance to *Verticillium* *dahliae*: genetics to genomic selection

Xiaojun Zhang^1,2#^ , Shiming Liu^1,2#^, Peng Wu^1,2^, Wanying Xu^1,2^, Dingyi Yang^1,2^, Yuqing Ming^1,2^, Shenghua Xiao^1^, Weiran Wang^1,3^, Jun Ma^3^, Xinhui Nie^4^, Zhan Gao^1^, Junyuan Lv^1,2^, Fei Wu^1,2^, Zhaoguang Yang^1^, Baoxin Zheng^1,2^, Ping Du^1,2^, Jiangmei Wang^1,2^, Hao Ding^1^, Jie Kong^3^, Alifu Aierxi^3^, Yu Yu^5^, Wei Gao^6^, Zhongxu Lin^1^, Chunyuan You^1,2^, Keith Lindsey^7^, Nataša Štajner^8^, Maojun Wang^1,2^, Jiahe Wu^9^, Shuangxia Jin^1,2^*, Xianlong Zhang^1,2^*, Longfu Zhu^1,2^*

^1^National Key Laboratory of Crop Genetic Improvement, Huazhong Agricultural University, Wuhan 430070, China.

^2^Hubei Hongshan Laboratory, Wuhan 430070, China.

^3^Institute of Economic Crops, Xinjiang Academy of Agricultural Sciences, Urumqi 830091, China.

^4^College of Agriculture, Shihezi University, Shihezi 832003, China.

^5^Cotton Research Institute, Xinjiang Academy of Agriculture and Reclamation Science, Shihezi 832000, China.

^6^State Key Laboratory of Cotton Biology, Henan University, Kaifeng 475001, China.

^7^Department of Biosciences, Durham University, Durham DH1 3LE, UK.

^8^Biotechnical Faculty, University of Ljubljana, Ljubljana 1000, Slovenia.

^9^State Key Laboratory of Plant Genomics, Institute of Microbiology, Chinese Academy of Sciences, Beijing 100101, China

^#^These authors contributed equally: Xiaojun Zhang, Shiming Liu

*Correspondence: [jsx@mail.hzau.edu.cn](mailto:jsx@mail.hzau.edu.cn) (Shuangxia Jin), [xlzhang@mail.hzau.edu.cn](mailto:xlzhang@mail.hzau.edu.cn) (Xianlong Zhang), [lfzhu@mail.hzau.edu.cn](mailto:lfzhu@mail.hzau.edu.cn) (Longfu Zhu).


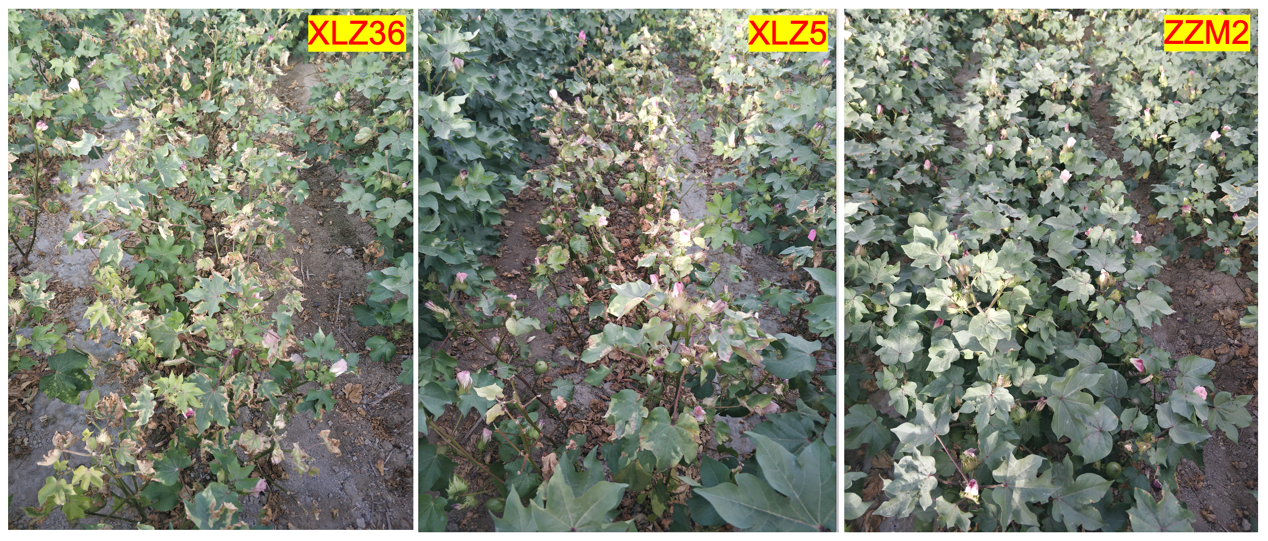


**Figure S1** **Cotton accessions represent the occurrence phenotypes in fields affected by *V. dahliae*.** The images were taken in Korla in 2018, and the materials from left to right are labeled as XLZ36, XLZ5, and ZZM2. ZZM2 was used as the disease-resistant control accessions, while XLZ36 served as the disease-susceptible control accessions. XLZ36, Xinluzao 36; XLZ5, Xinluzhong 5; ZZM2, Zhongzhimian 2.


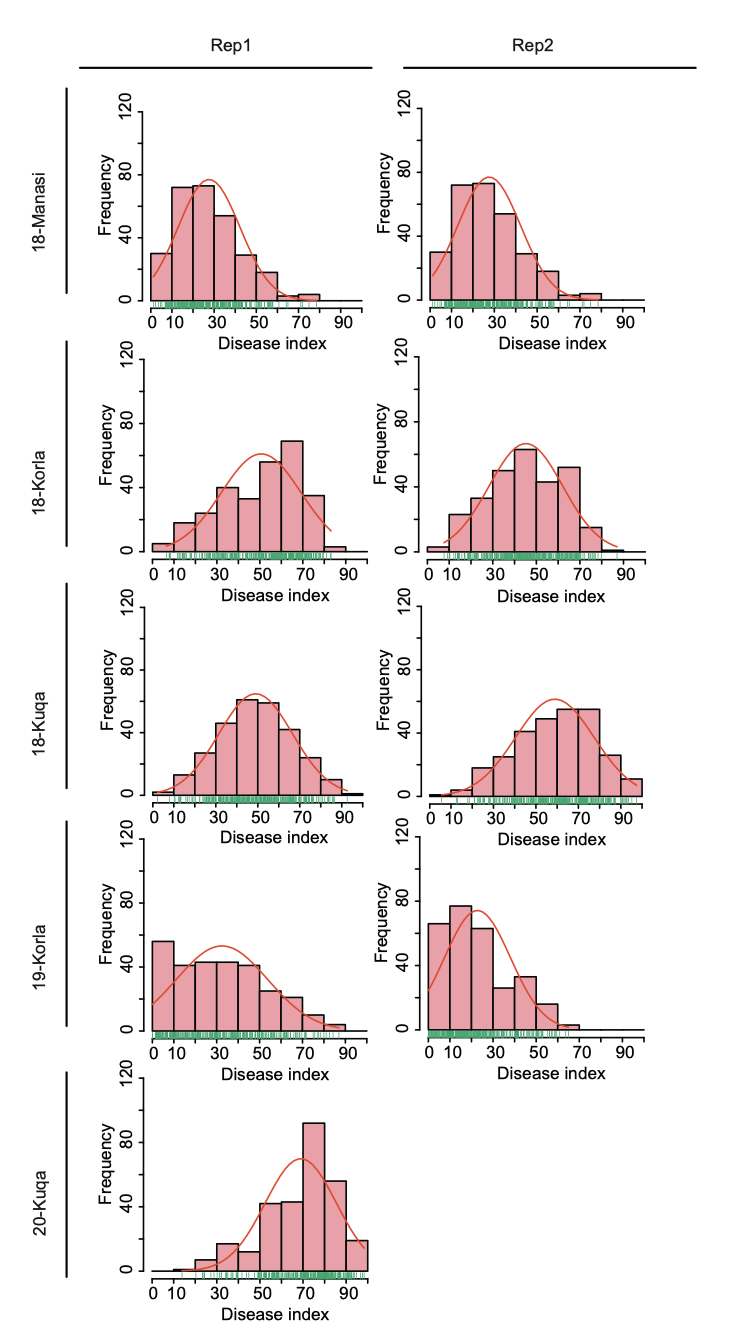


**Figure S2** **Histogram of the frequency distribution of DI of 290 upland cotton** **accessions in five independent environments.** The x-axis represents disease index, the y-axis represents the number of cotton accessions, with a step size of 10. The green line on the x-axis represents the distribution of each accession, and the red line represents the fitted normal density curve. Rep1 and Rep2 represent the two replicates in this environment, respectively. 18, year 2018; 19, year 2019; 20, year 2020.


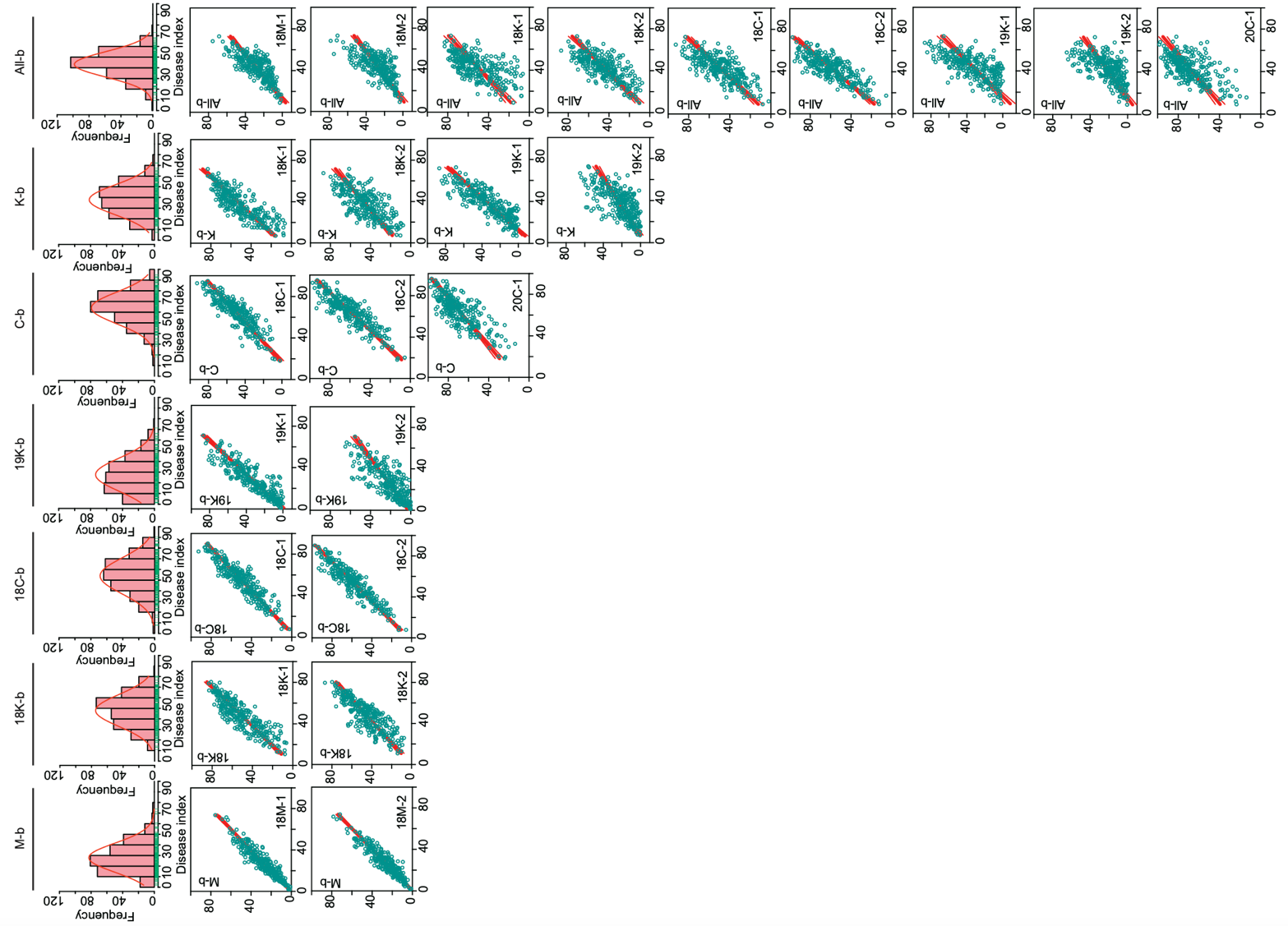


**Figure S3** **Correlation between DI in different environments and the BLUE adjusted DI.** The top row displays a frequency histogram of DI after applying the BLUE treatment, with the red line representing the fitted normal density curve. Below the histogram shows the raw DI set and its correlation used in the BLUE treatment, where the straight line denotes simple linear regression, and the solid and dashed lines correspond to mean and error, respectively. M, Manasi; K, Korla; C, Kuqa; b, BLUE; 18, year 2018; 19, year 2019; 20, year 2020.


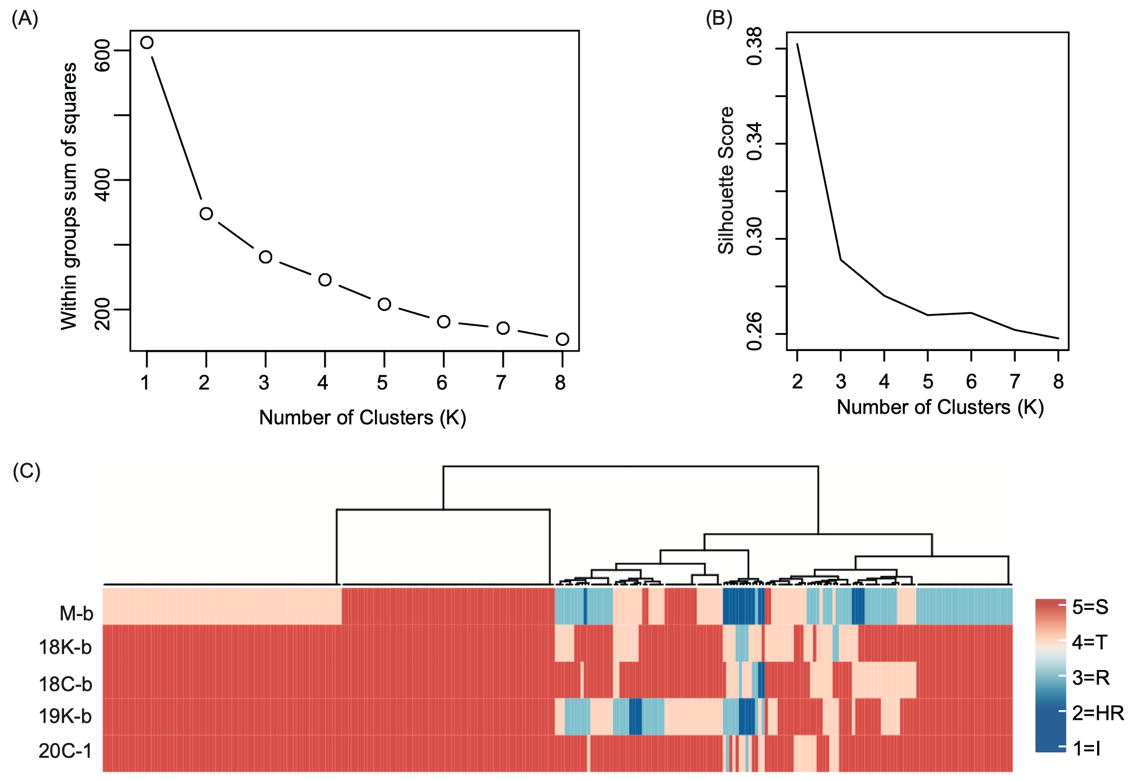


**Figure S4 Cluster analysis of DI of 290 upland cotton accessions across five environments.** (A−B) Represent the K values determined for clustering based on SSE (sum of the squared error) and silhouette coefficient, respectively. The x-axis represents the number of clusters (K value). (C) Heatmaps of DI for 290 cotton accessions in five independent environments. 1−5 assignment values respectively represent immune (I), highly resistant (HR), resistant (R), tolerant (T), susceptible (S). M, Manasi; K, Korla; C, Kuqa; b, BLUE; 18, year 2018; 19, year 2019; 20, year 2020.


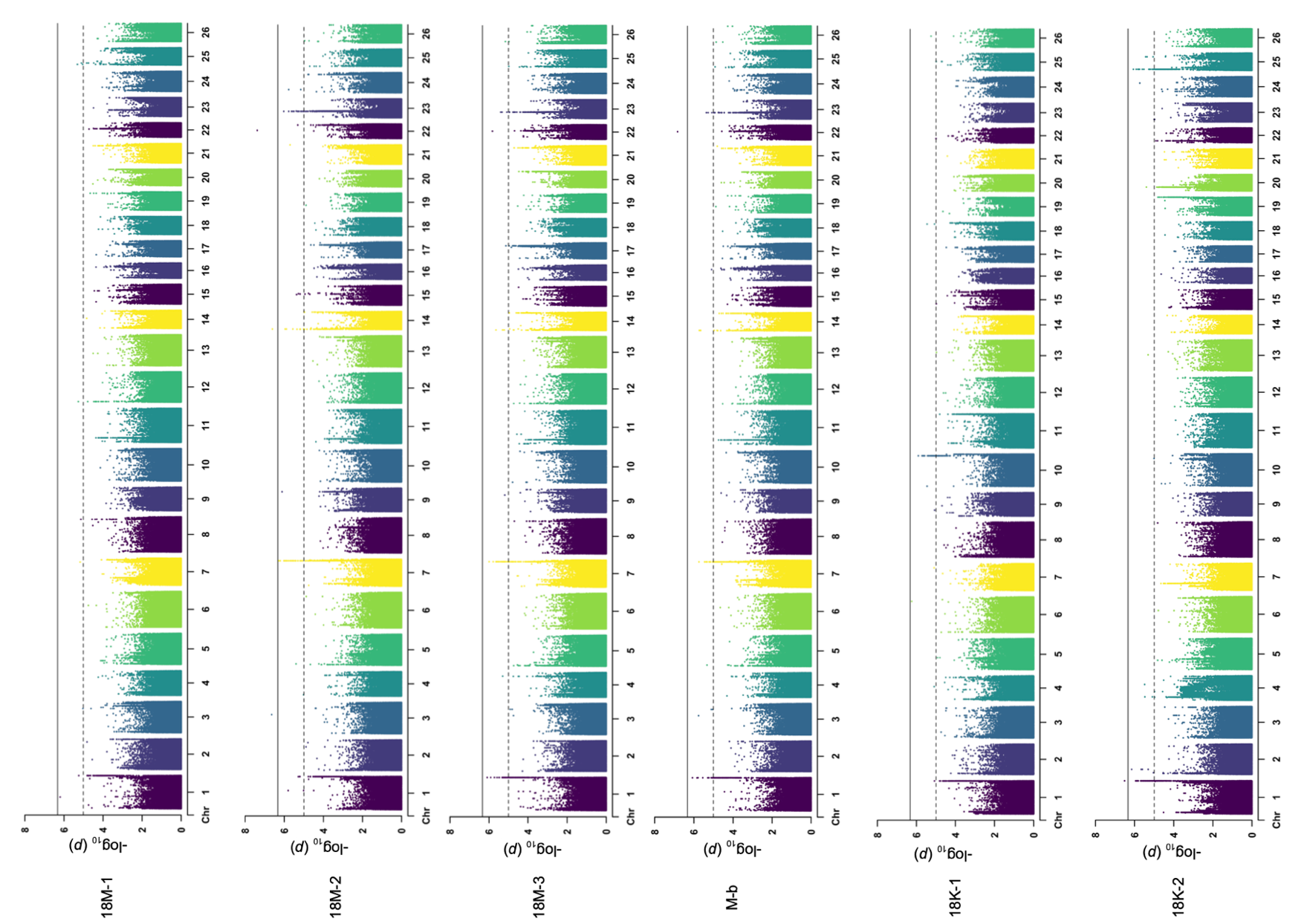


**Figure S5 Manhattan plot of 20 DI sets in 290 accessions**. The label on the left side of each Manhattan plot is the name of the DI set, and the x-axes represents the cotton chromosomes A01−A13, D01−D13, and the y-axes represents the -log_10_ (*p*). Significance thresholds of *p* = 4.65E-05 and *p* = 1E-05. M, Manasi; K, Korla; C, Kuqa; b, BLUE; 18, year 2018; 19, year 2019; 20, year 2020.


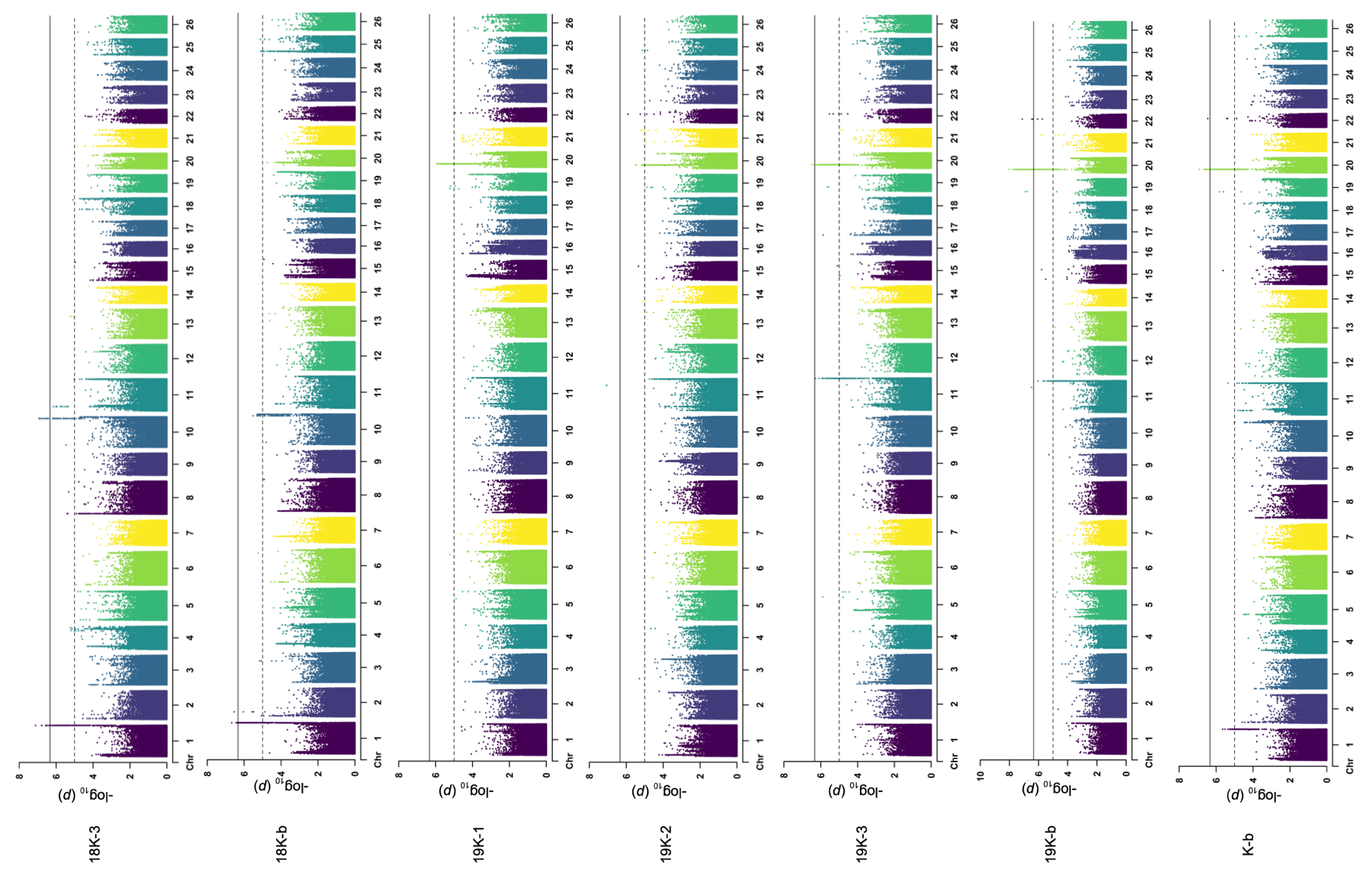


**Figure S5 Continued.**


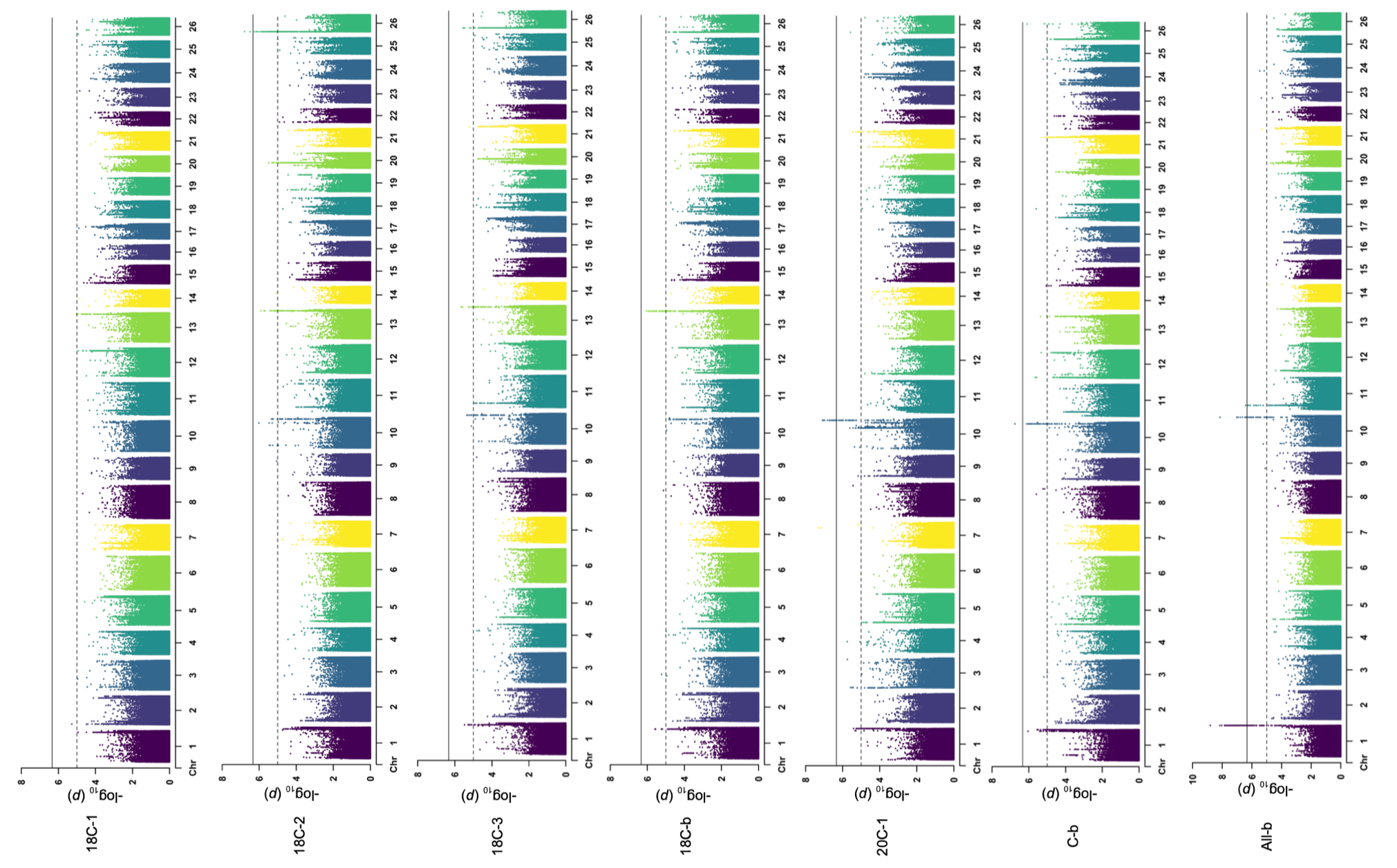


**Figure S5 Continued.**

**
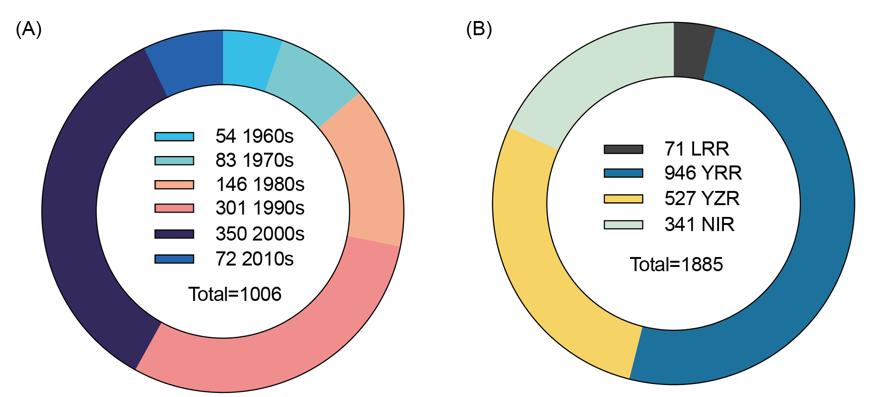
**

**Figure S6 Collected information on the breeding era and geographic distribution of the CDCs.** (A) Breeding era information collected for CDCs, with the numbers before the labels indicating the number of varieties. (B) Geographic distribution information collected for CDCs, with the numbers before the labels indicating the number of varieties. CDCs, Chinese descendible cultivars; LRR, Liao River region; YRR, Yellow River region; YZR, Yangtze River region; NIR, Northwest region.


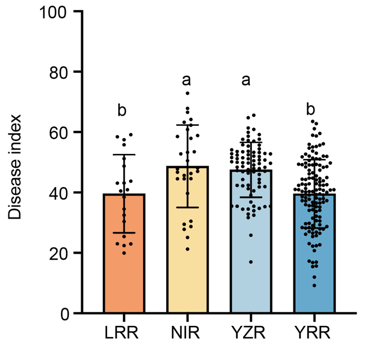


**Figure S7 Comparison of DI among 290 upland cotton accessions from the four major cotton-growing regions**. The y-axis represents all-b DI set. The letters indicate the statistical test after the *t*-test (p < 0.05). LRR, Liao River region; YRR, Yellow River region; YZR, Yangtze River region; NIR, Northwest region.


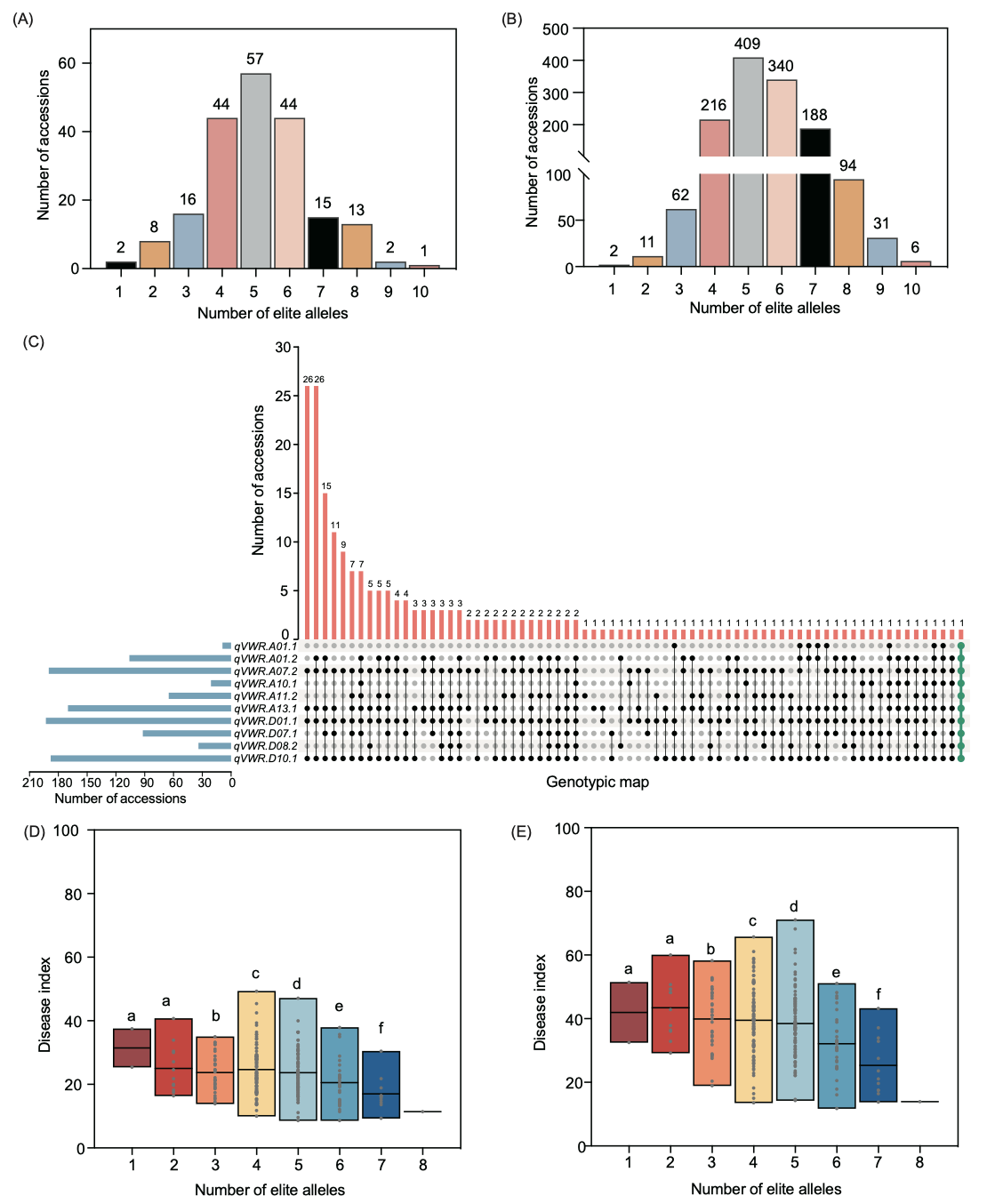


**Figure S8 Pyramiding analysis of 10 Lsnp^R^s among the upland cotton accessions.** (A) Distribution of 10 Lsnp^R^s in 290 upland cotton accessions. The x-axis represents the summation of cotton accessions carrying 1−10 Lsnp^R^. (B) Distribution of 10 Lsnp^R^s in 2033 CDCs. The x-axis represents the summation of cotton accessions carrying 1−10 Lsnp^R^. (C) The genotype upset of 290 upland cotton accessions on 10 Lsnp^R^s. The solid circles represent resistant genotypes (Lsnp^R^) at the Lsnp, with the cultivar Zhongzhimian 2 (carrying 10 Lsnp^R^s) highlighted by green-filled circles, and bar charts represent the quantity of such accessions. (D−E) Distribution of DI in other natural cotton accessions carrying different numbers of Lsnp^R^. The x-axis represents cotton accessions carrying 1−10 Lsnp^R^. The y-axis represents DI. The letters indicate the statistical test after the *t*-test (*p* < 0.001). The DIs used for D−E are DI at 20 dpi and 25 dpi, respectively. CDCs, Chinese descendible cultivars;


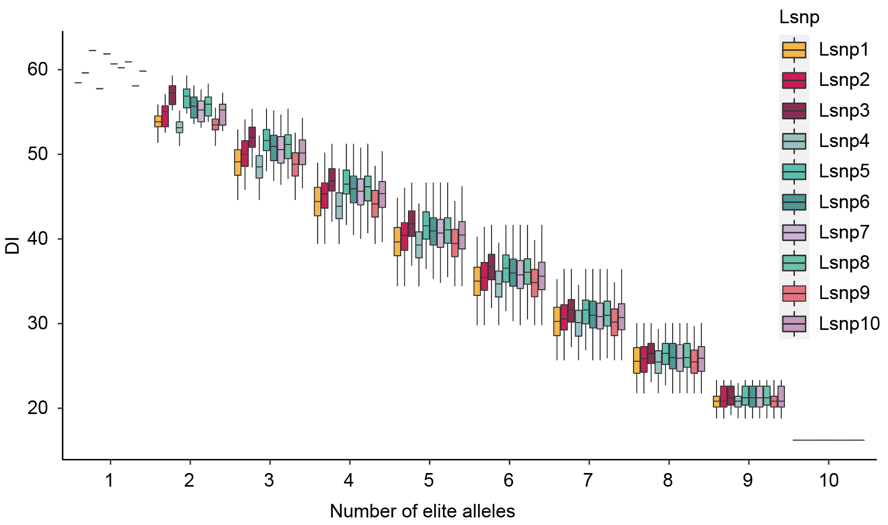


**Figure S9 The DI distribution of simulated varieties carrying different numbers of Lsnp^R^**. The x-axis represents cotton accessions carrying 1−10 Lsnp^R^. The boxes of different colors represent Lsnp1−10 from left to right.


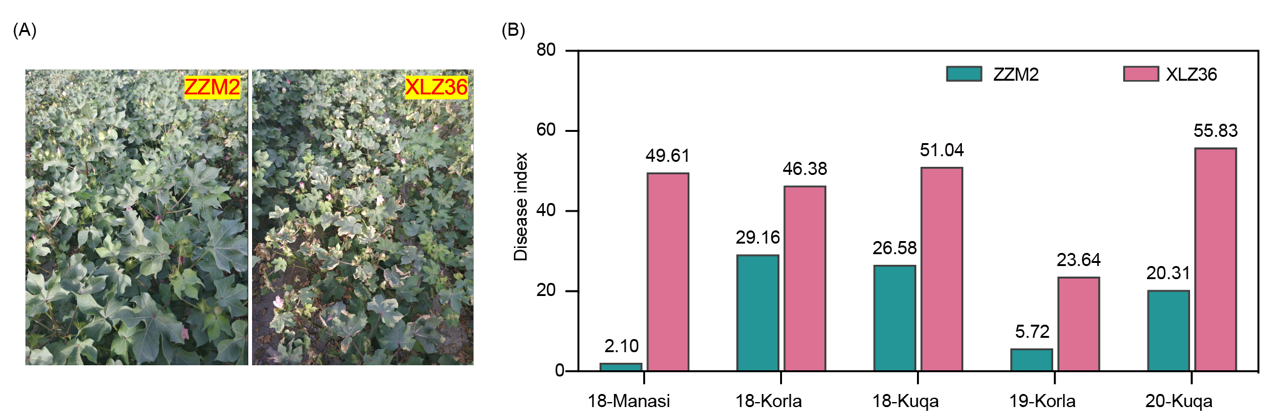


**Figure S10** **Field resistance phenotypes of the parents used for constructing the artificial population**. (A) The images in field resistance phenotypes of the parents were taken in Korla in 2019. (B) Histogram of the DI of parents in different environments. ZZM2, Zhongzhimian 2; XLZ36, Xinluzao 36; 18, year 2018; 19, year 2019; 20, year 2020.


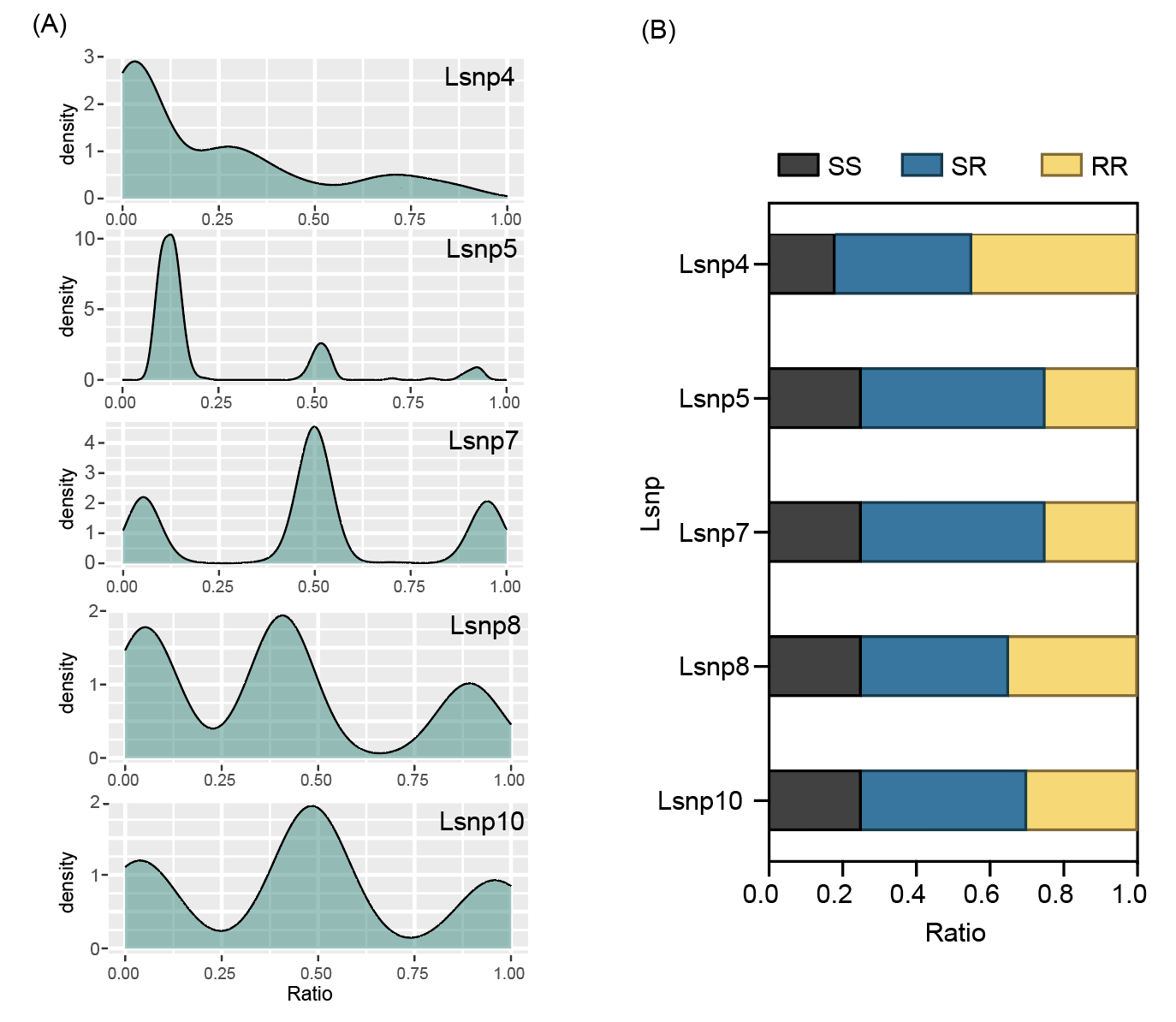


**Figure S11 Density plot of high-throughput sequencing genotypes in 272 F_2_ individuals at 5 Lsnps**. (A) Density plots of 272 F_2_ individuals were determined based on high-throughput sequencing of 5 Lsnp, and the genotype of each material was determined based on the proportions of the three peaks corresponding to the horizontal axis. (B) The genotyping principle of the 5 Lsnp markers was determined based on the proportion of reads obtained from sequencing, the x-axis represents the interval values of read proportions for the three genotypes. R, resistance; S, susceptibility.


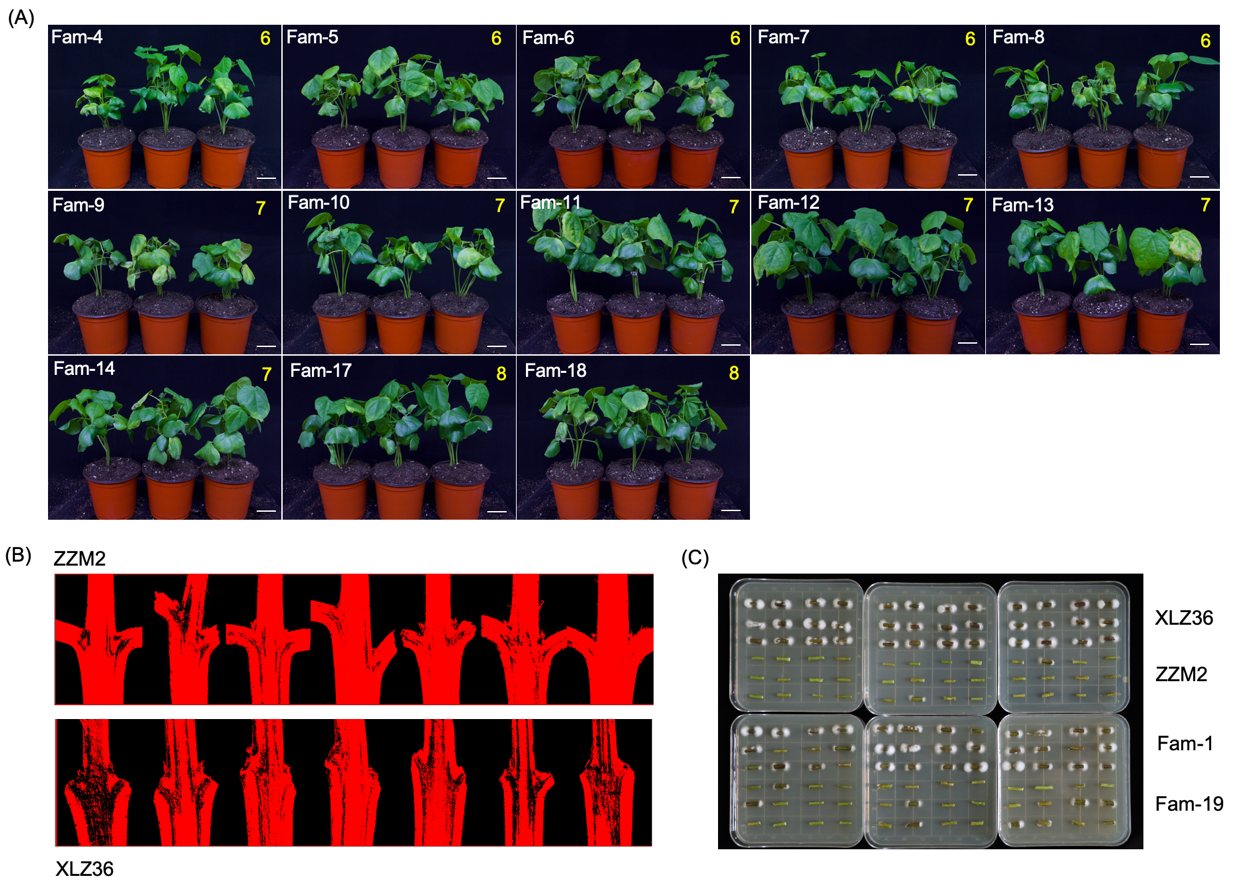


**Figure S12 Pyramiding analysis of Lsnp^R^s among F_2:3_ lines with exclusion of six extreme F_2:3_ lines.** (A) The images showing the disease phenotype. Photographed at 13 days post-inoculation. The yellow number in the top right corner indicates the number of Lsnp^R^ carried by the lines. Scale bar, 3 cm. (B) Extracting the area of lesions from the images of representative materials ZZM2 and XLZ36 after stem sectioning. (C) Fungal recovery assay showing the growth of hypha around inoculated plants on potato dextrose agar (PDA) medium. ZZM2, Zhongzhimian 2; XLZ36, Xinluzao 36.


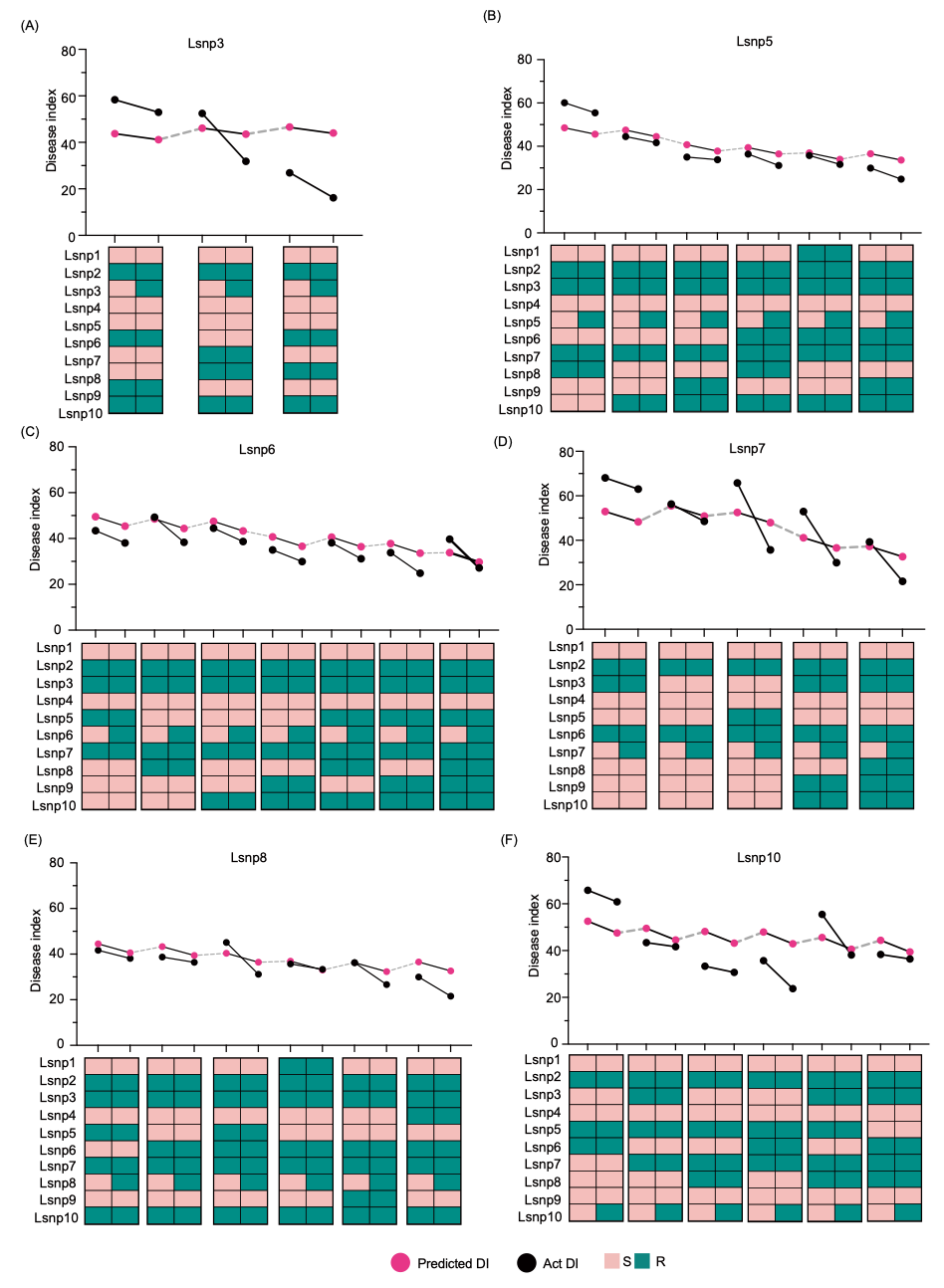


**Figure S13 The effect of enhancing VW resistance in existing cotton materials after transformation from Lsnp^S^ to Lsnp^R^.** (A−F) represent the DI distribution between the Lsnp^S^ (pink) series cotton varieties and Lsnp^R^ (green) series cotton varieties at Lsnp3, Lsnp5, Lsnp6, Lsnp7, Lsnp8 and Lsnp10. The genotypic map (below x-axis) shows haplotypes across accessions, with the upper line graph indicating mean DI per haplotype. The black dots represent the actual DI, the magenta dots represent the predicted DI by the molecular disease index calculator.


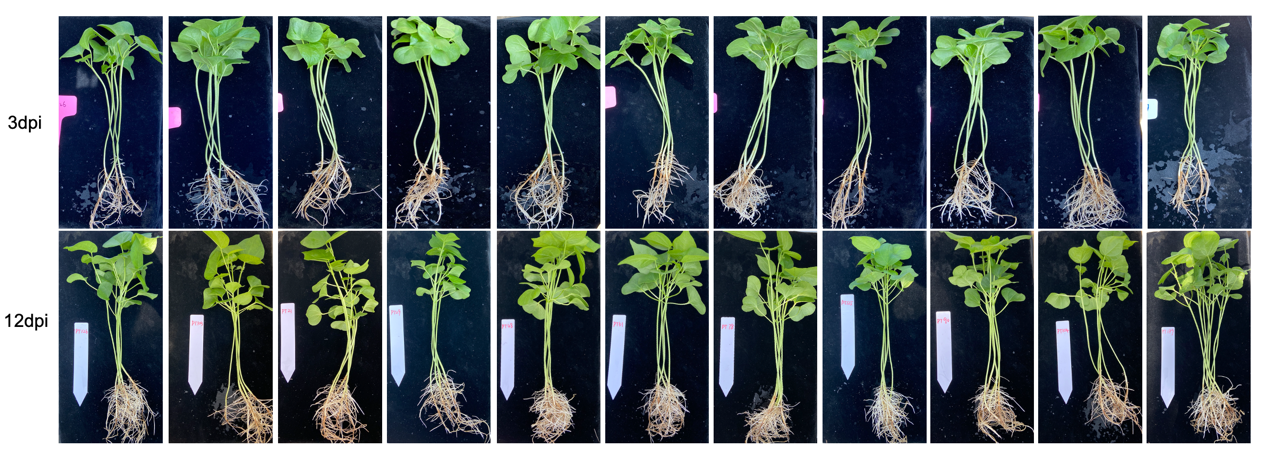


**Figure S14 Sampling design of the population transcriptome at 3 and 12 days post-inoculation with *V. dahliae*.**


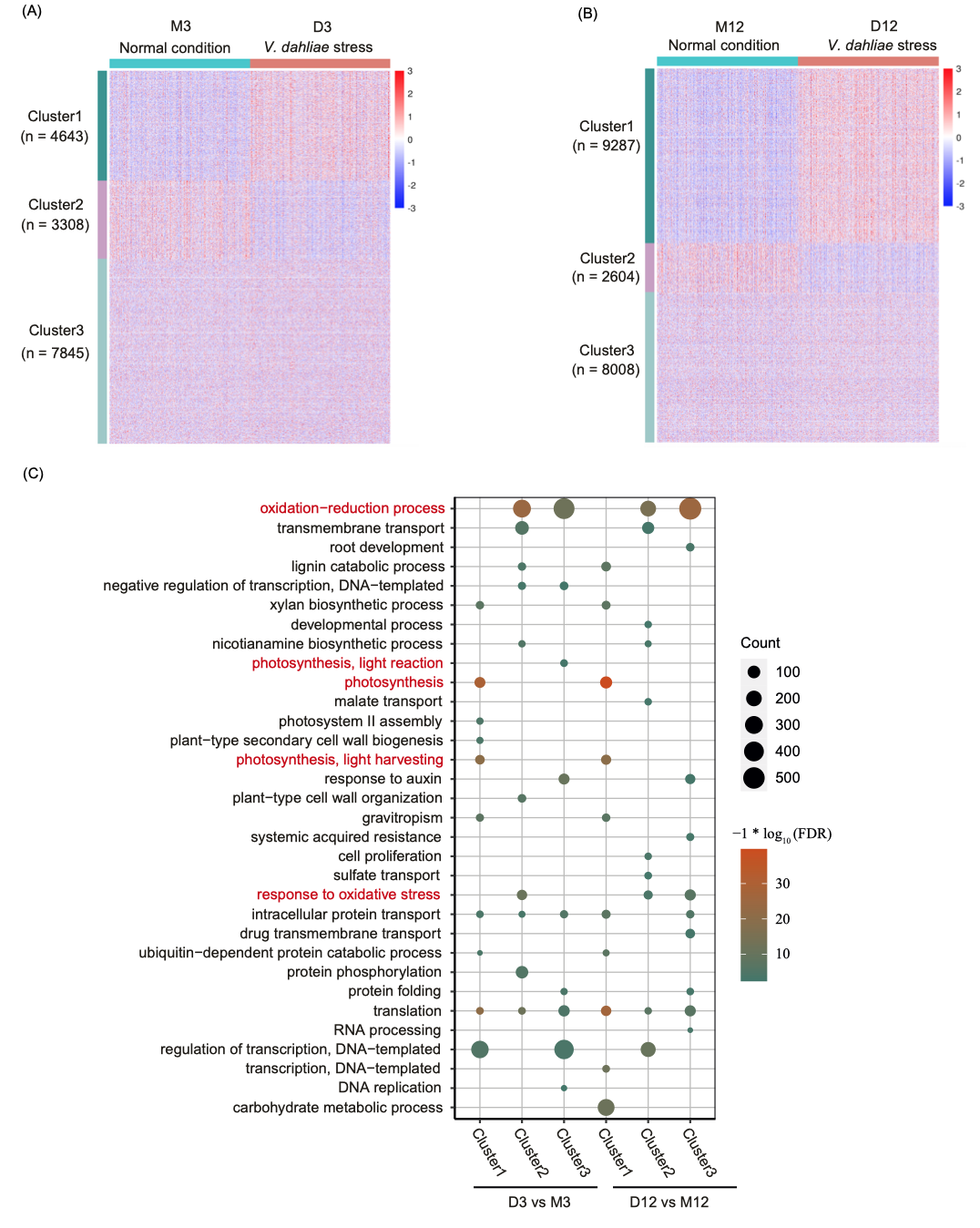


**Figure S15 Expression pattern clustering and GO enrichment analysis of differentially expressed genes (DEGs) in response to *V. dahliae* infection.** (A−B) Expression heatmap of DEGs between control and 3 days post-inoculation (A) and12 days post-inoculation (B). (C) Top 10 Go terms of biological process for each cluster genes. M3, 3 days after mock treatment; D3, 3 days post-inoculation; M12, 12 days after mock treatment; D12, 12 days post-inoculation.


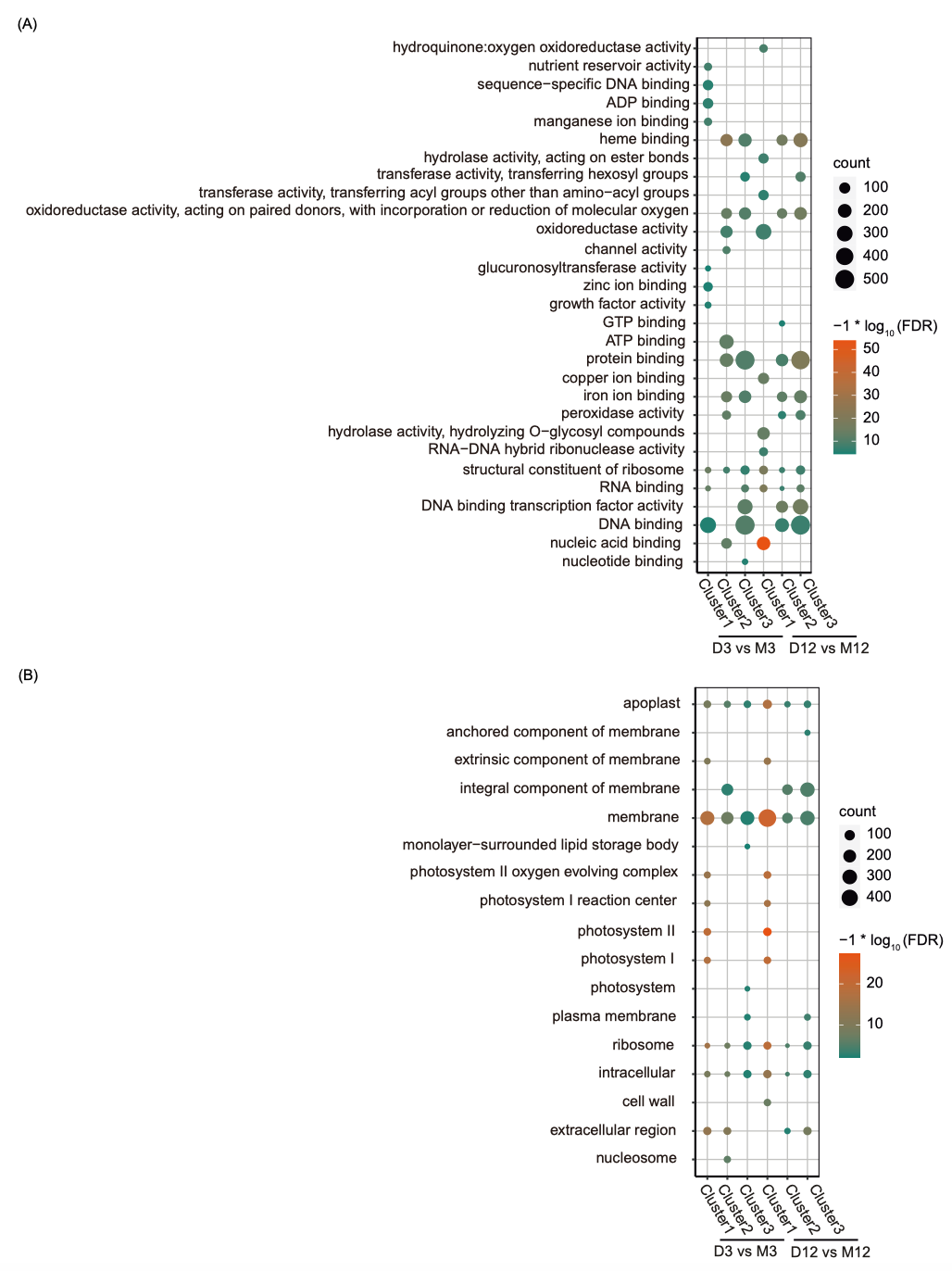


**Figure S16 GO enrichment analysis of differentially expressed genes (DEGs) in response to *V. dahliae* infection involved in molecular function and cell cellular**. (A−B) Top 10 Go terms of molecular function (A) and cell cellular (B) for the DEGs in each cluster.


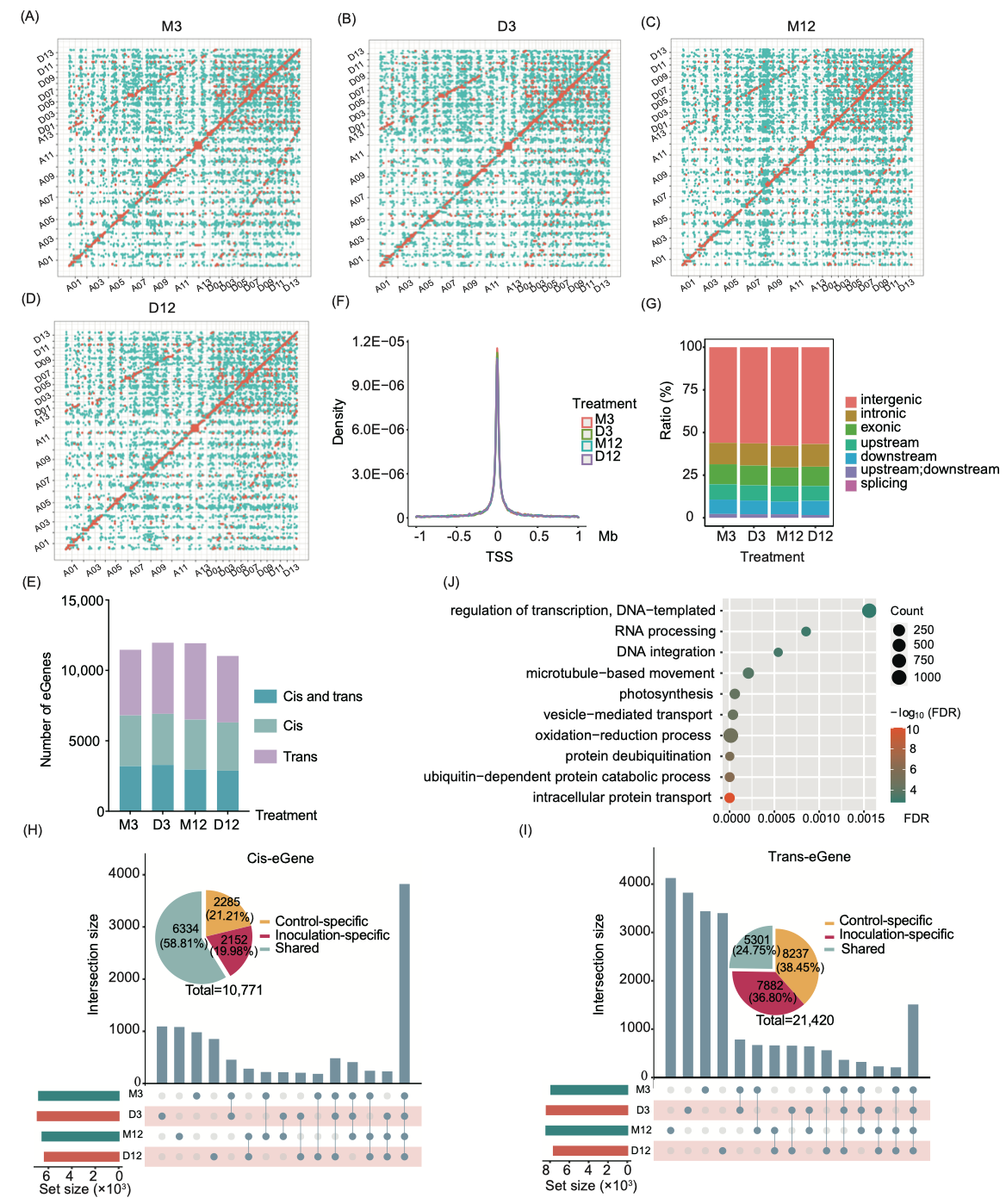


**Figure S17 Distribution and annotation of eQTLs and functions of eGenes involved.** (A−D) The distribution of eQTLs and their corresponding eGenes across 26 chromosomes for M3(A), D3(B), M12(C), and D12(D). X-axis represent the position of SNP in each chromosome and Y-axis represent the position of eGene. The red dots represent eQTLs with an r^2^ value exceeding 0.2. (E) The number of eGenes regulated by cis-eQTL, trans-eQTL or both. (F) Distribution of the distances between cis-eQTLs and the transcription start sites (TSS) of their associated eGenes. Upstream and downstream regions are defined as 2000 bp from the gene. (G) Genomic annotation of cis-eQTLs under various inoculation treatments. (H−I) Upset plot and pie chart displaying the number and ration of commonly and specifically cis-eGenes (H) and trans-eGenes (I) between mock and v991 inoculation treatments. The term “mock-specific” refers to eGenes that are exclusively identified in at least one stage of the mock treatment, while "inoculation-specific" indicates eGenes identified solely in at least one stage of the v991 treatment. (J) GO enrichment analysis of all inoculation-related eGenes, including mock-specific and inoculation-specific eGenes. TOP 10 signiﬁcantly enriched biological process GO terms are show. M3, 3 days after mock treatment; D3, 3 days post-inoculation; M12, 12 days after mock treatment; D12, 12 days post-inoculation.


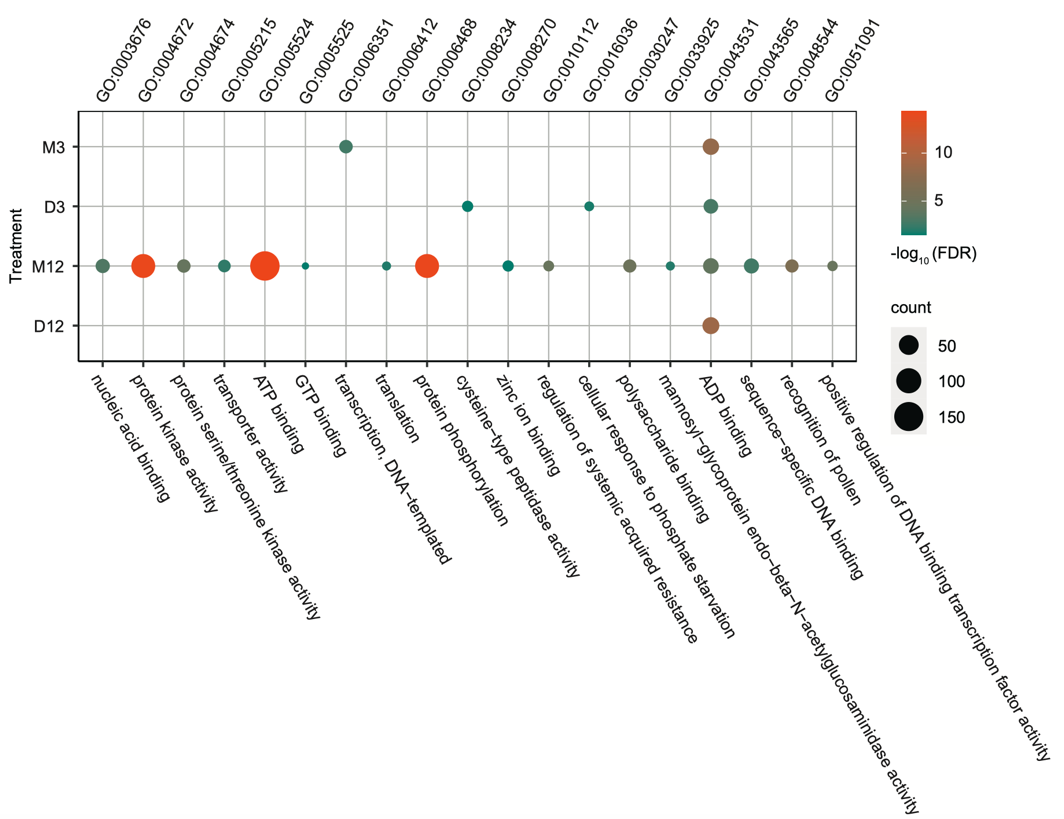


**Figure S18 GO enrichment analysis of genes in co-expressed gene modules associated with QTLs.** All significant (FDR-corrected *p* value ≤ 0.05) GO terms for each treatment were shown. M3, 3 days after mock treatment; D3, 3 days post-inoculation; M12, 12 days after mock treatment; D12, 12 days post-inoculation.

**
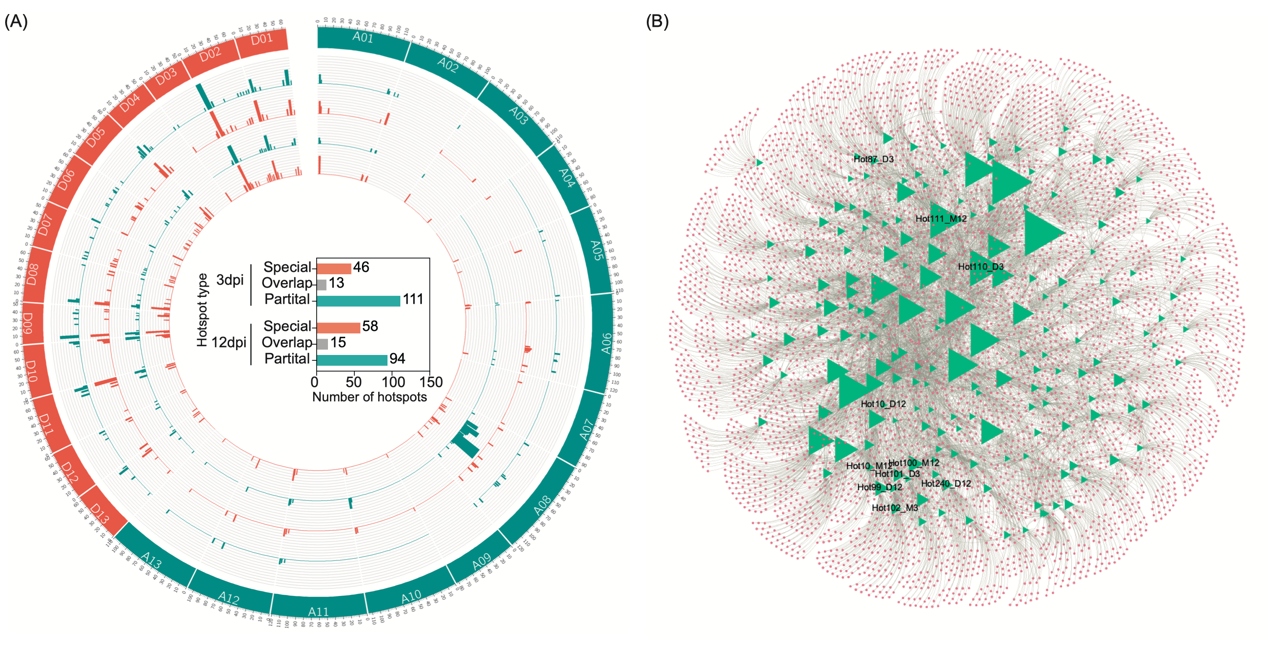
**

**Figure S19 Distribution of trans-eQTL hotspots and gene regulatory networks correlated with *V. dahliae* resistance.** (A) The genomic distribution of eQTL hotspots among four treatments. From outer to inner, they are M3, D3, M12, and D12. The bars of the histogram in the circle indicates the number of genes regulated by hotspots. the inner upset graph showcases the specific and shared eQTL hotspots among the four treatments. (B) Networks of eQTL hotspot and its regulated genes exhibiting functions similar to *V. dahliae*-responsive genes. Triangle node represents eQTL hotspots and circular represent eGenes. M3, 3 days after mock treatment; D3, 3 days post-inoculation; M12, 12 days after mock treatment; D12, 12 days post-inoculation.


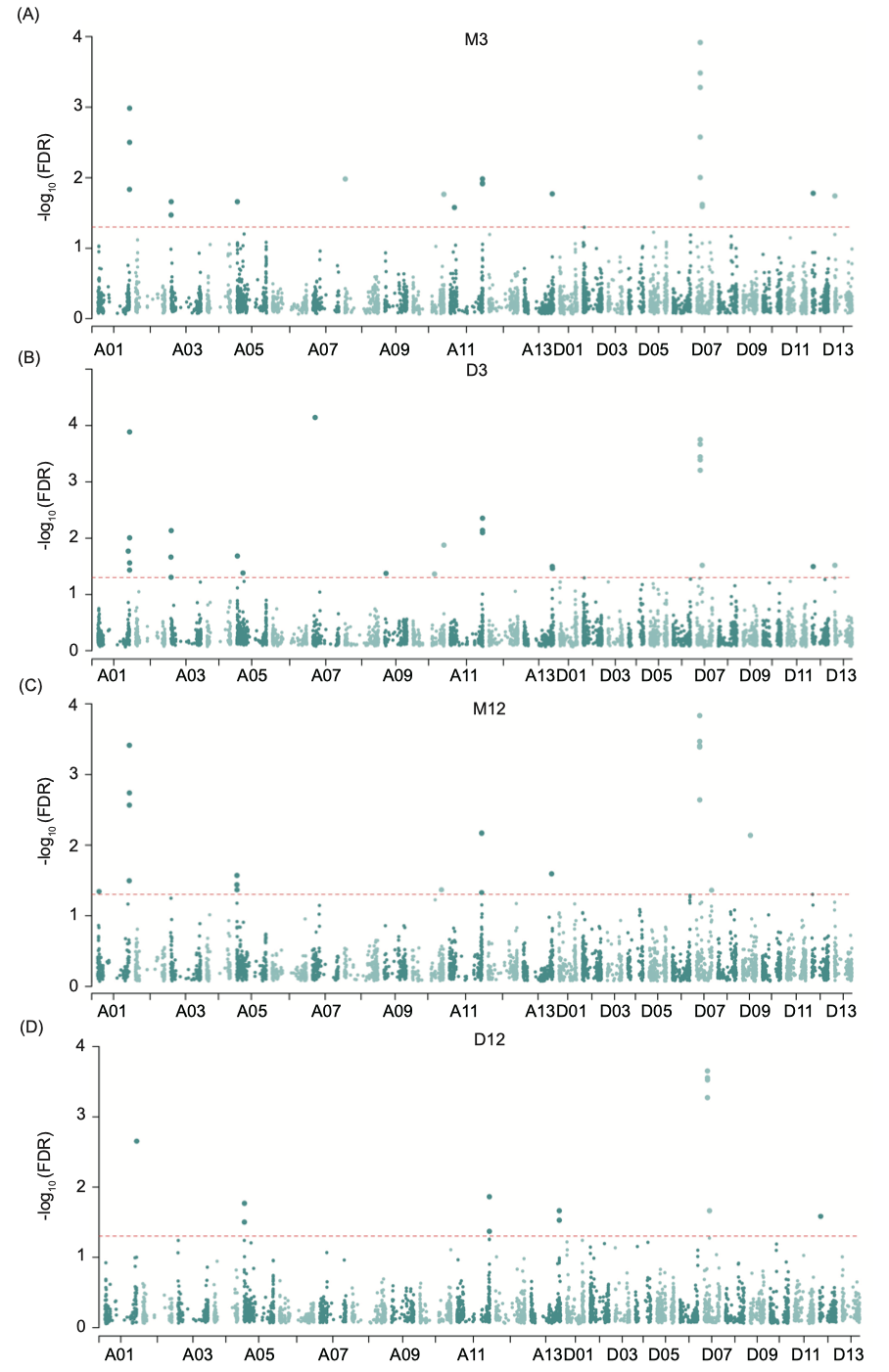


**Figure S20 Individual Manhattan plots of the transcriptome-wide association study analysis (TWAS) for four treatments.** (A−D) Manhattan plots of TWAS for M3 (A), D3 (B), M12 (C) and D12 (D)**,** respectively. Each point represents an individual gene, The red dashed line represents the signiﬁcance threshold -log_10_ (FDR) = 0.05. M3, 3 days after mock treatment; D3, 3 days post-inoculation; M12, 12 days after mock treatment; D12, 12 days post-inoculation.


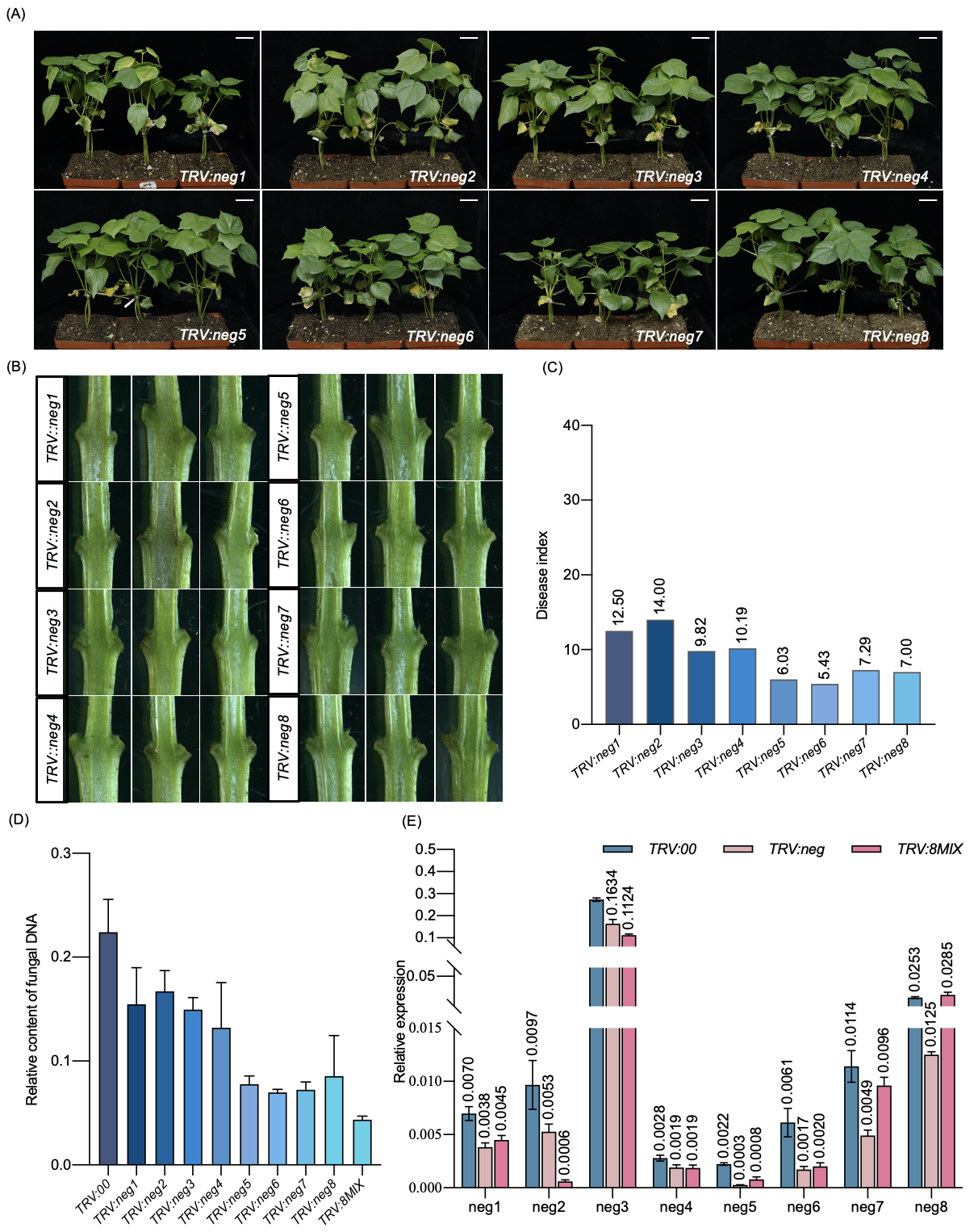


**Figure S21 The individual knockdown of eight negatively regulated genes resulted in improved cotton resistance to *V. dahliae****.* (A) Disease symptoms of cotton plants with gene knockdown. Scale bar, 3 cm. (B) Vascular bundle coloration in longitudinal sections of *TRV:negx* cotton stems. (C) Disease index of *TRV:negx* cotton plants. (D) Measurement of fungal biomass in *TRV:negx* cotton stems. (E) Relative expression of target genes in *TRV:00* and *TRV:negx* leaves.

**
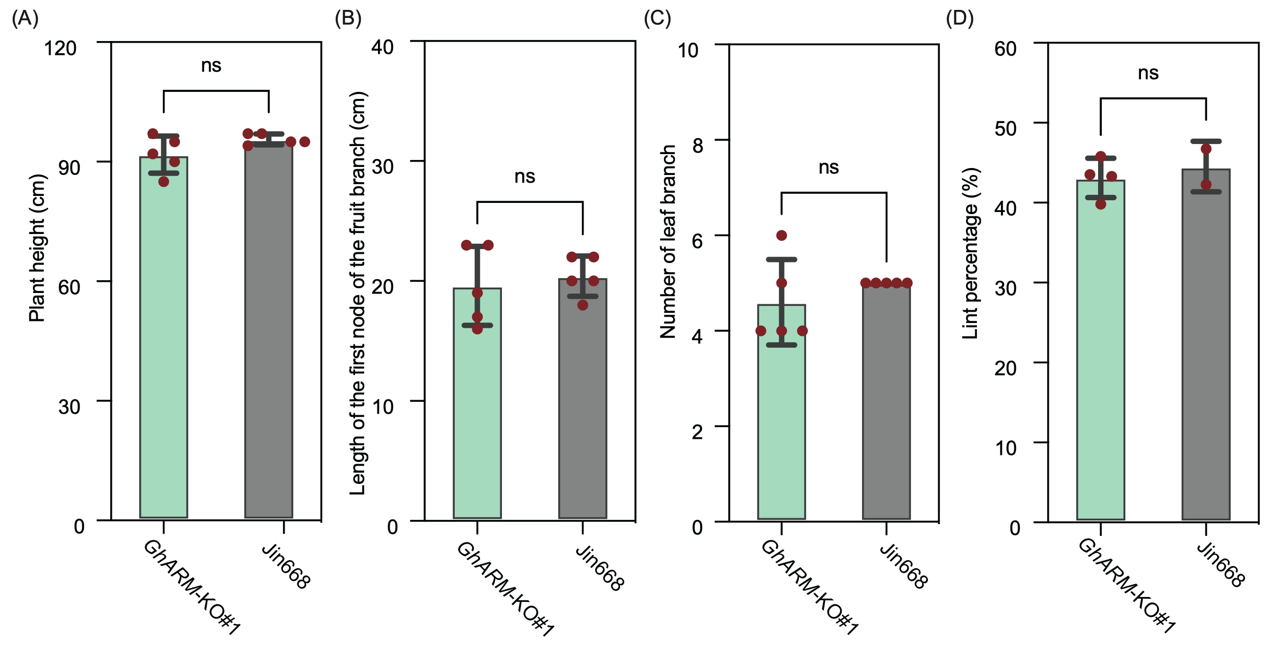
**

**Figure S22 Field evaluation for agronomic trait of Jin668 and *GhARM* knockout cotton plants**. (A) Plant height. (B) Length of the first node of the fruit branch. (C) Number of leaf branch. (D) Lint percentage. The statistical test after the *t*-test.


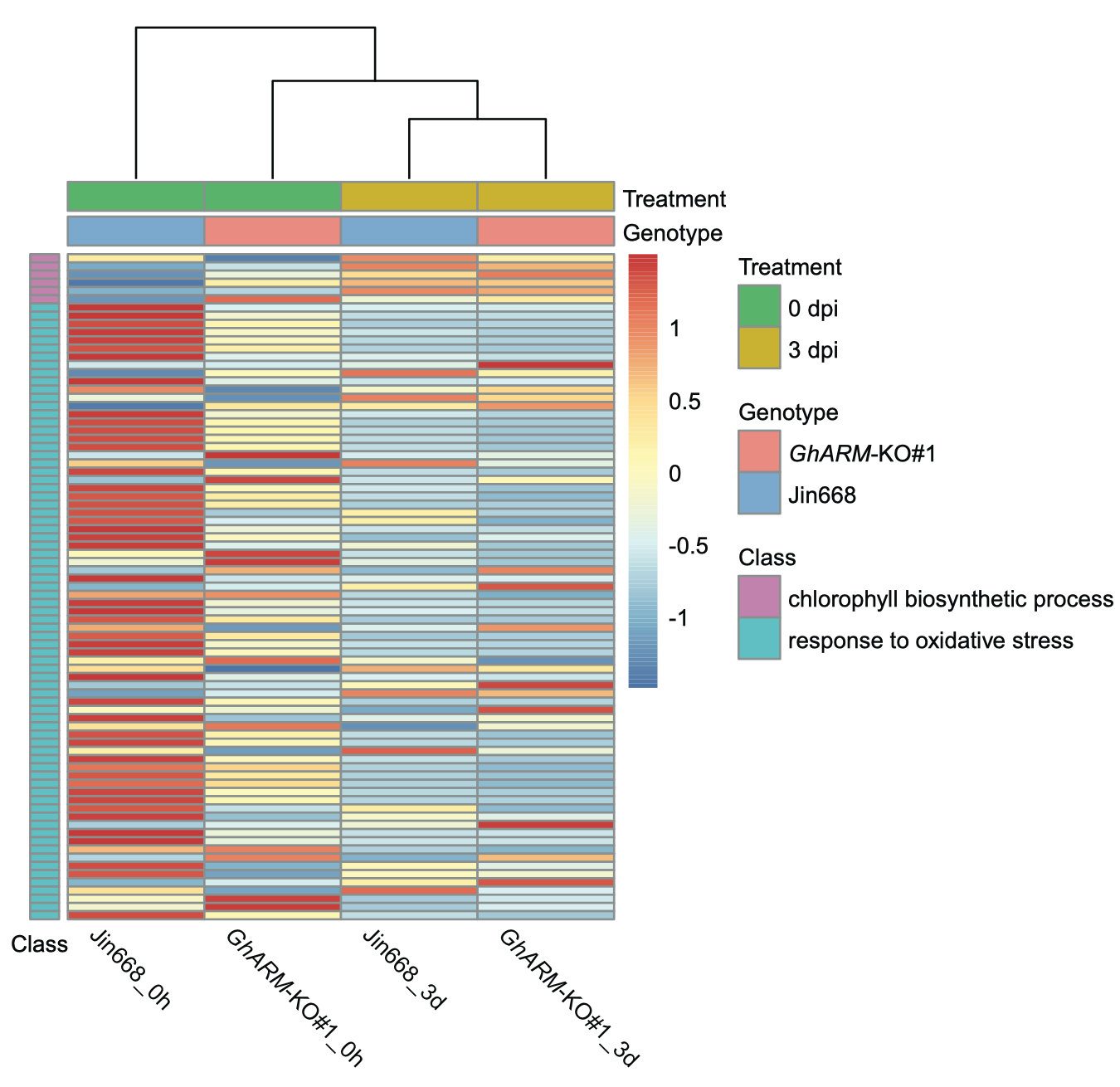


**Figure S23 Heatmap for the expression of genes involved in chlorophyll biosynthetic process and response to oxidative stress.**

**
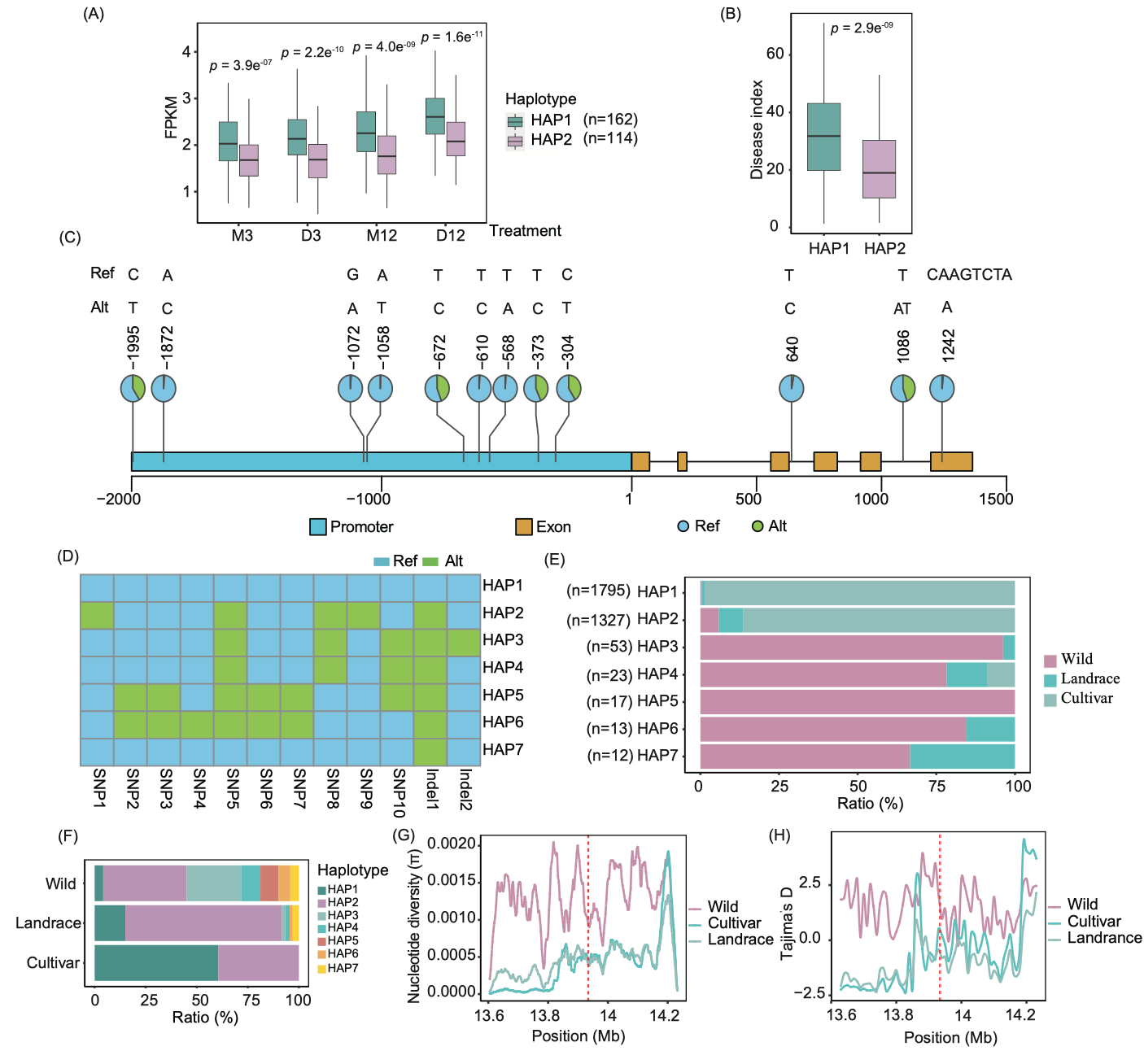
**

**Figure S24 Natural variation and selection footprint analysis of *GhARM*.** (A) Comparison of gene expression levels of *GhARM* among individuals with different haplotypes in 290 cotton accessions. (B) Comparison of disease index for 19K-b among individuals with the two main haplotype of *GhARM* in 290 cotton accessions. The statistical significance for A and B was calculated using two-sided Wilcoxon rank sum test. In box plots of A and B, central line represents median, box limits indicate first and third quartiles. (C) Natural variations of *GhARM* in 4180 cotton accessions. (D) Heatmap of seven haplotypes for *GhARM*. (E−F) Distribution and proportions of seven haplotypes in *G. hirsutum* (wild), *G. hirsutum* (landrace) and *G. hirsutum* (cultivars). (G−H) Nucleotide diversity (π) analysis and Tajima’s D test across the 300 kb genomic region flanking the *GhARM* gene.

**
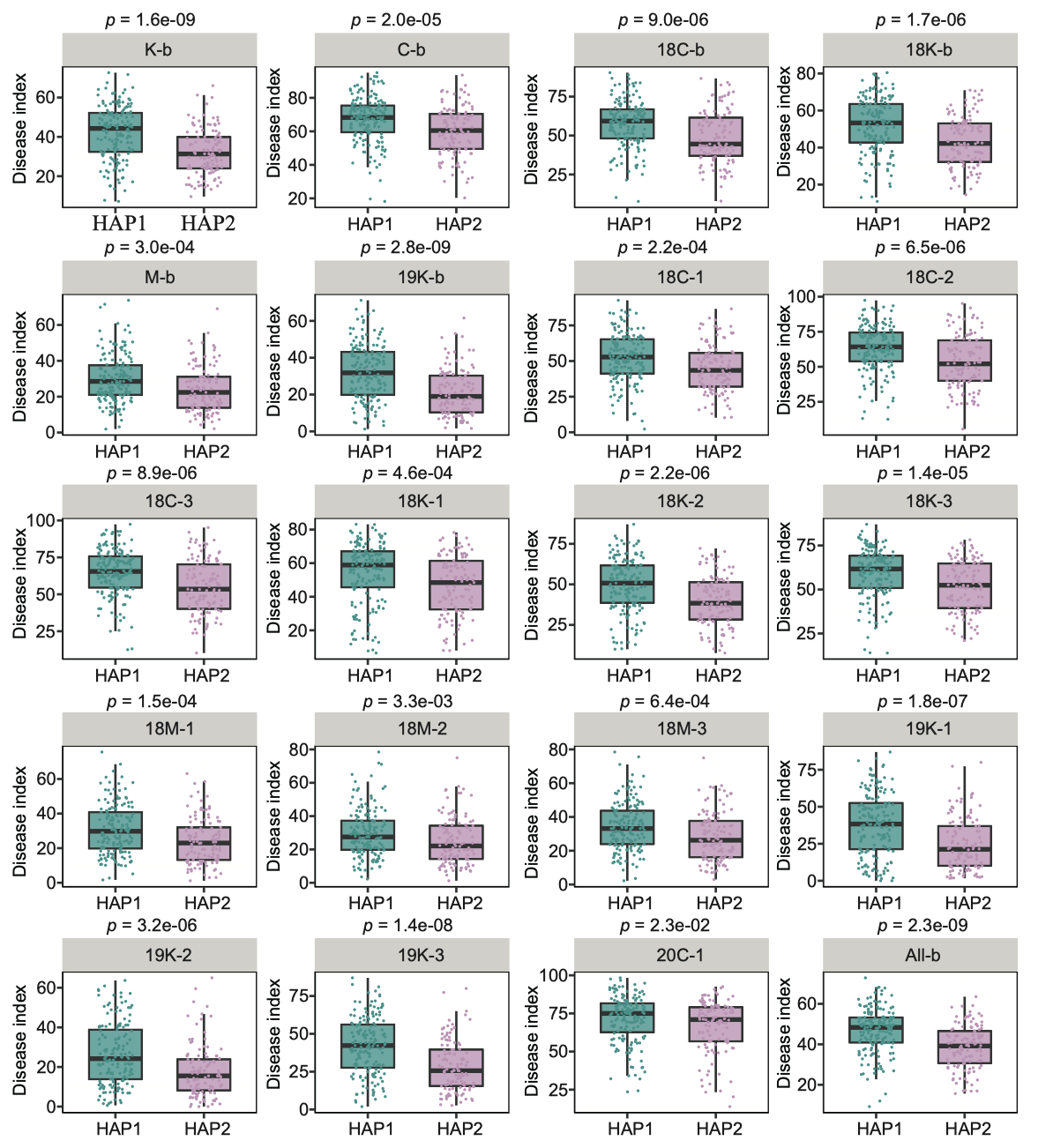
**

**Figure S25 Comparison of DI for all 20 DI sets among individuals with the two main haplotypes of *GhARM* in 290 cotton accessions**. Each box plot represents one set DI. The statistical significance was calculated by Wilcoxon rank sum test (center line, median; box limits, first and third quartiles).
